# Genetically programmed alternative splicing of NEMO mediates an autoinflammatory disease phenotype

**DOI:** 10.1172/JCI128808

**Published:** 2022-03-15

**Authors:** Younglang Lee, Alex W. Wessel, Jiazhi Xu, Julia G. Reinke, Eries Lee, Somin M. Kim, Amy P. Hsu, Jevgenia Zilberman-Rudenko, Sha Cao, Clinton Enos, Stephen R. Brooks, Zuoming Deng, Bin Lin, Adriana A. de Jesus, Daniel N. Hupalo, Daniela G.P. Piotto, Maria T. Terreri, Victoria R. Dimitriades, Clifton L. Dalgard, Steven M. Holland, Raphaela Goldbach-Mansky, Richard M. Siegel, Eric P. Hanson

**Affiliations:** 1 Immunodeficiency and Inflammatory Disease Unit and; 2 Immunoregulation Section, Autoimmunity Branch, National Institute of Arthritis and Musculoskeletal and Skin Diseases (NIAMS), NIH, Bethesda, Maryland, USA.; 3 Indiana University School of Medicine, Wells Center for Pediatric Research, Indianapolis, Indiana, USA.; 4 Immunopathogenesis Section, Laboratory of Clinical Immunology and Microbiology (LCIM), National Institute of Allergy and Infectious Diseases, NIH, Bethesda, Maryland, USA.; 5 Department of Biostatistics, Indiana University, School of Medicine, Indianapolis, Indiana, USA.; 6 Biodata Mining and Discovery Section, Office of Science and Technology, NIAMS and; 7 Translational Autoinflammatory Diseases Section (TADS), LCIM, National Institute of Allergy and Infectious Diseases, NIH, Bethesda, Maryland, USA.; 8 The American Genome Center, Collaborative Health Initiative Research Program, Uniformed Services University of the Health Sciences, Bethesda, Maryland, USA.; 9 Escola Paulista de Medicina/Universidade Federal de São Paulo, São Paulo, Brazil.; 10 Division of Infectious Diseases, Immunology & Allergy University of California Davis Health, Sacramento, California, USA.; 11 Department of Anatomy, Physiology & Genetics, Uniformed Services University of the Health Sciences, Bethesda, Maryland, USA.; 12 Novartis Institutes for BioMedical Research WSJ, Basel, Switzerland.

**Keywords:** Genetics, Immunology, Inflammation, Genetic diseases, Innate immunity, Signal transduction

## Abstract

Host defense and inflammation are regulated by the NF-κB essential modulator (NEMO), a scaffolding protein with a broad immune cell and tissue expression profile. Hypomorphic mutations in inhibitor of NF-κB kinase regulatory subunit gamma (*IKBKG*) encoding NEMO typically present with immunodeficiency. Here, we characterized a pediatric autoinflammatory syndrome in 3 unrelated male patients with distinct X-linked *IKBKG* germline mutations that led to overexpression of a NEMO protein isoform lacking the domain encoded by exon 5 (NEMO-Δex5). This isoform failed to associate with TANK binding kinase 1 (TBK1), and dermal fibroblasts from affected patients activated NF-κB in response to TNF but not TLR3 or RIG-I–like receptor (RLR) stimulation when isoform levels were high. By contrast, T cells, monocytes, and macrophages that expressed NEMO-Δex5 exhibited increased NF-κB activation and IFN production, and blood cells from these patients expressed a strong IFN and NF-κB transcriptional signature. Immune cells and TNF-stimulated dermal fibroblasts upregulated the inducible IKK protein (IKKi) that was stabilized by NEMO-Δex5, promoting type I IFN induction and antiviral responses. These data revealed how *IKBKG* mutations that lead to alternative splicing of skipping exon 5 cause a clinical phenotype we have named NEMO deleted exon 5 autoinflammatory syndrome (NDAS), distinct from the immune deficiency syndrome resulting from loss-of-function *IKBKG* mutations.

## Graphical abstract

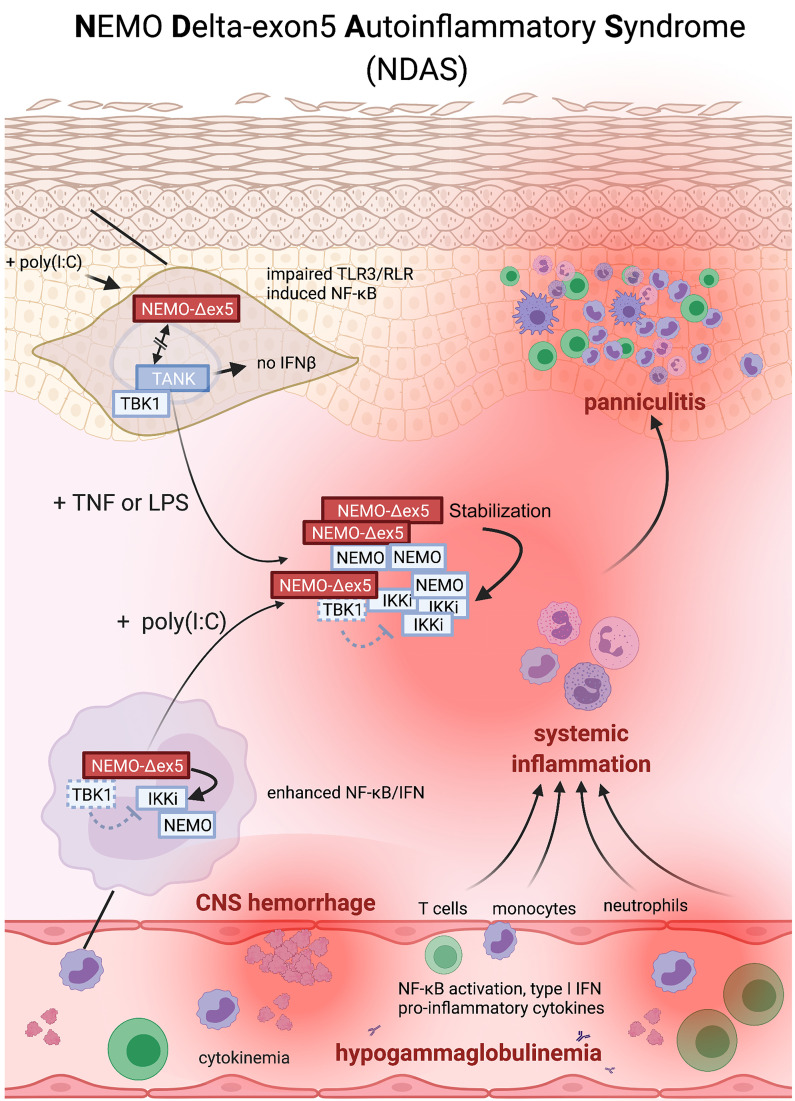

## Introduction

The NF-κB essential modulator (NEMO/IKK-γ) transduces signals in response to the stimulation of pattern recognition receptors, the TNF receptor superfamily, and lymphocyte antigen receptors, and thus plays a central role in both innate and adaptive immunity. NEMO plays a critical role in signaling downstream of nucleic acid sensing by TLR3 and the RIG-I–like receptors (RLRs), which induce IFNs essential to the antiviral immune response. NEMO relays signals from these receptors by activating both IRF3 and the NF-κB family of transcription factors. It activates these transcription factors by serving as an essential nonenzymatic regulator of the canonical IKK kinases (IKK-α and IKK-β); the IKK-related kinases (TBK1 and inducible IKK [IKKi]); and other components of these signaling pathways, such as TRAF-associated NF-κB regulator (TANK) and A20. Through NEMO and its co-adaptor TANK, these IKKs activate transcription by phosphorylation of IRF3 and the p65 and p105 subunits of NF-κB in response to TLR3 and RLR stimulation (1, 2). This pathway is distinct from the noncanonical IKK pathway triggered in response to activation of TNFR superfamily members leading to p52 and RELB NF-κB subunit transactivation. In addition to activating TLR3 and RLR signaling, IKKi and TBK1 can also downregulate canonical IKK activity (3, 4), but the role of cross-talk between alternative and canonical NF-κB activation pathways in human disease has not been described. Sensing of viral and endogenous nucleic acids induces IFNs essential to the host antiviral immune response, a process dependent on NEMO and the canonical IKK complex. Thus, RLR, TLR3, and NEMO deficiency all lead to susceptibility to viral infection and serious sequelae, such as herpes simplex virus type 1 encephalitis (5). By contrast, activating mutations in nucleic acid sensing pathways lead to inflammatory disease (6), although how NEMO couples with the IKK-related kinases in different cell types to maintain homeostasis, balancing inflammation and immunity, remains unclear.

NEMO mutation frequently results in an X-linked clinical disorder called anhidrotic ectodermal dysplasia (EDA), which is due to impaired NF-κB activation in response to EDA receptor signaling during development (7). Most NEMO mutations are hypomorphic and lead to a combined immunodeficiency and susceptibility to encapsulated bacteria, DNA viruses, and atypical mycobacteria (8, 9). For example, deletion mutations affecting the N-terminus impair association of NEMO with the catalytic subunits IKK-α/β and lead to fatal herpes virus infection, whereas deletion mutations of the C-terminus (ΔCT-NEMO) lead to an autoinflammatory syndrome combined with immunodeficiency (10–13). Hence, whereas certain mutant forms of NEMO lead to disease due to loss of NF-κB activation, other forms in which inflammatory disease predominates may be secondary to impaired regulation of proinflammatory pathways. Isoforms of NEMO resulting from alternative splicing have been described in cell lines and patients (10, 14–16), but the molecular mechanisms by which these isoforms lead to disease are not completely understood.

Here, we describe the clinical and molecular autoinflammatory disease features associated with 3 mutations that lead to the expression of a NEMO protein isoform lacking the domain encoded by exon 5, termed NEMO-Δex5. We found that NEMO-Δex5 disrupted type I IFN expression in response to TLR-3 and/or RLR stimulation. However, TNF costimulation enhanced the responsiveness of this pathway in skin fibroblasts, inducing the expression of the atypical IKK kinase, IKKi, which we showed here to be specifically stabilized in the presence of NEMO-Δex5. In contrast to dermal fibroblasts, patient peripheral blood monocytes, macrophages, and T cells exhibited robust proinflammatory cytokine production and an excess of IFN production, which was associated with decreased viral replication. Thus, genetically programmed expression of the NEMO-Δex5 protein isoform leads to an inflammatory disease phenotype. Our data suggest that altered signaling characteristics conferred by high expression levels of the NEMO-Δex5 isoform may have a role in host immunity and inflammatory disease.

## Results

### Expression of an alternative NEMO isoform causes systemic autoinflammatory disease.

**Figure 1:**
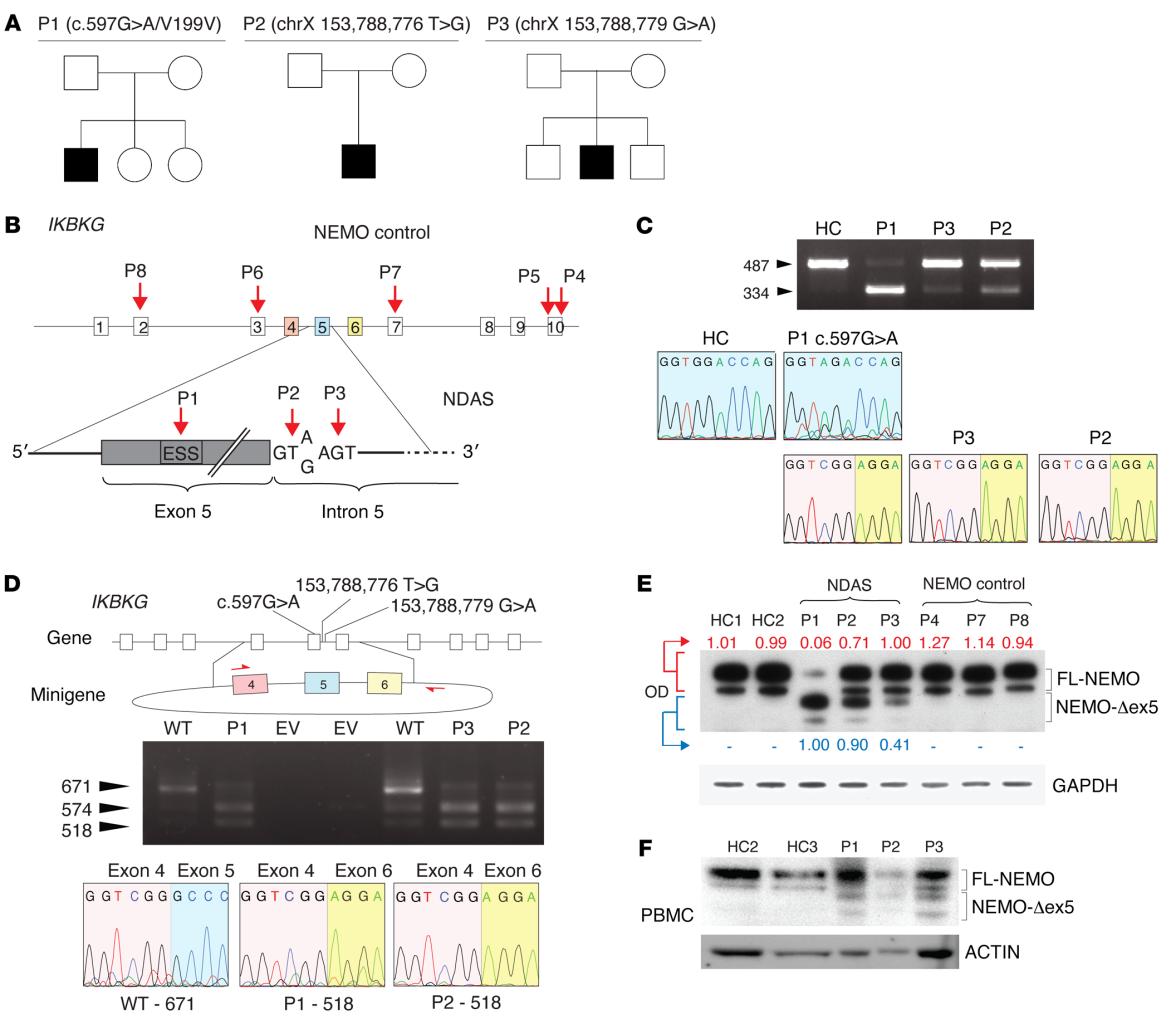
A synonymous exonic mutation and 2 intronic splice donor mutations mediate alternative splicing of *IKBKG* and lead to severe autoinflammatory disease. (**A**) Pedigree and clinical features of developmental and inflammatory diseases in patients P1–P3. (**B**) Schema of mutations identified in patients P1–P3 and other NEMO mutants. Note exon coloring used for **C** and **D**. Patient P4 has the well-described NEMO-C417R zinc finger hypomorphic mutation. I DNA gel electrophoresis of *IKBKG* cDNA from P1, P2, P3, and healthy control (HC) dermal fibroblasts. Chromatograms of Sanger-sequenced FL isoforms from P1 and HCs and the variant isoform from P1–P3. (**D**) Schema of minigene spanning *IKBKG* exons 4–6 containing the reference sequence or patient mutations. DNA electrophoresis of cDNA and sequence traces are shown as In **C**. The 574 bp fragment contains noncoding intronic sequence in addition to a fragment of exon 5. WT = *IKBKG* minigene containing reference sequence. (**E**) Western blot of NEMO from patients P1–P3 and NEMO control patient skin fibroblasts, optical densitometry of FL-NEMO relative to loading control is shown in red, of NEMO-Δex5 in blue. (**F**) Western blot of PBMCs and whole cell lysate from patients P1, P2, and P3 and 2 healthy controls (HCs).

We evaluated a 5-year-old male patient (P1) who first presented with elevated serum markers of systemic inflammation and with tissue- and organ-specific inflammation starting at a few weeks of age (described in detail in Methods and Supplemental Figure 1, A–I; supplemental material available online with this article; https://doi.org/10.1172/JCI128808DS1). Briefly, initial disease manifestations included fevers, a nodular skin rash, and elevated liver enzymes that progressed to include sterile panniculitis, optic neuritis, and chorioretinitis. There was no familial history of a similar disease (Figure 1A). Extensive evaluations excluded an infectious etiology and targeted Sanger sequencing for known periodic fever genes, including *MEFV*, *NOD2/CARD15*, *NLRP3*, and *TNFRSFIA*, as well as whole-exome sequencing, failed to identify likely causative inherited or de novo rare missense or nonsense candidate variants. At 1 year of age, the eruption of conical-shaped teeth raised the suspicion of EDA. Although the absence of infections made classic EDA with immune deficiency (EDA-ID) unlikely, the combination of immune dysregulation coupled with abnormal tooth development suggested a possible underlying functional defect in NEMO, IκBα, or a functionally related gene, which could disrupt ectodysplasin signaling and produce the observed dental phenotype. The phenotype prompted targeted sequencing of inhibitor of NF-κB kinase regulatory subunit gamma (*IKBKG*), the gene encoding NEMO. A synonymous c.597G>A substitution within exon 5 in *IKBKG* was identified. In silico analyses of the c.597G>A mutation predicted disruption of an exonic splicing enhancer and an exon-identity element in exon 5, which could promote alternative splicing of this exon (17, 18) (see Methods for analysis details). Indeed, analysis of whole-blood RNA-Seq data revealed an alternative splicing ratio (see Methods) of 56.7% in patient P1, whereas in healthy controls the ratio was 8.5% (95% CI 6.1%–11.0%). This transcript had previously been identified in cancer cell lines and healthy control CD4^+^ T cells (15). We further examined alternative *IKBKG* splicing in a large cohort of patients with autoinflammatory disease and found alternative splicing ratios of 64.3% and 40.1% in 2 additional patients, P2 and P3 (Supplemental Figure 1J). Sanger sequencing of DNA from these 2 male patients revealed 2 de novo single nucleotide changes in the intronic splicing regulatory domain of exon 5, thus identifying 2 additional patients with related genetic polymorphisms and similar clinical features (Figure 1, A and B, and Table 1). Whole-genome sequences from all 3 patients, P1, P2, and P3, revealed no other likely disease-causing candidate variants (Methods). *IKBKG* sequencing of maternal DNA indicated that all 3 mutations were de novo.

To assess the specificity of alternative splicing in cells from patients with splice-site mutations, we performed RNA-Seq analysis on PBMCs of 3 immunodeficient NEMO patients with known loss-of-function mutations, patients P4, P5, and P7. We identified no increase in alternative splicing compared with healthy controls (Supplemental Figure 1J). We next determined whether the alternative splicing in *IKBKG* was confined to hematopoietic immune cells. In patients P1, P2, and P3, a shorter NEMO mRNA splice variant was detected in fibroblasts, with variable levels of the full-length (FL) transcript. Sanger sequencing of the shorter NEMO variant confirmed the same 153 nucleotide deletion of exon 5 in all 3 patients, P1, P2, and P3 (Figure 1C). Sequence analysis of the long cDNA variant from P1 cells confirmed the synonymous exon 5 c.597G>A mutation that was identified by Sanger sequencing of genomic DNA (Figure 1C). Quantitation of this variant and of the FL transcript by qPCR indicated significantly higher ratios of mutant over FL cDNA in P1–P3 than in healthy controls (Supplemental Figure 1K). Because the splice variant is not captured in public databases, we analyzed whole-genomic variation data from more than 1150 healthy individuals with ethnic backgrounds similar to those of the patients (45.3% southern European, 24.3% pan-European, 18.2% western/northern European), and we failed to detect the 3 presumed pathogenic variants (Supplemental Figure 1L). To confirm that these mutations independently promote loss of exon 5 during mRNA splicing, we constructed a minigene containing *IKBKG* exons 4 to 6 (Figure 1D). Whereas transfection with a minigene containing the reference genomic sequence yielded a majority of FL transcript, transfection with minigenes containing mutant sequences from patients P1, P2, and P3 led to recovery of increased amounts of shortened transcript variants (Figure 1D). Verification by Sanger sequencing confirmed that the 518 bp amplicon contained sequence-skipping exon 5. The shortened transcript maintained the open reading frame, thus predicting expression of a protein lacking amino acids 174 through 224 of FL-NEMO. We therefore refer to this protein isoform as NEMO-Δex5. To test whether the splice variant generated a stable protein product, we analyzed NEMO protein expression in primary cells from patients P1, P2, and P3. In dermal fibroblasts from patient P1, we observed low levels of FL-NEMO protein expression and high levels of NEMO-Δex5, the identity of which we confirmed by mass spectroscopy (Figure 1E). Cells from patients P2 and P3 had lower levels of the mutant isoform and higher levels of FL-NEMO compared with P1 (Figure 1E). In PBMCs from patients P1, P2, and P3, we also detected NEMO-Δex5, which was not detectable in cells from healthy controls (Figure 1F). To understand the effect of NEMO-Δex5 mutation on NF-κB–mediated gene expression, we performed RNA-Seq on whole blood from patients P1–P3, 13 healthy controls, and 10 patients with NOMID, an IL-1–mediated autoinflammatory disease caused by gain-of-function mutations in *NLRP3*. Cells from patients P1–P3 exhibited a markedly enhanced NF-κB response expression profile compared with healthy controls and patients with NOMID (Supplemental Figure 1M). Together, these data demonstrated that these rare *IKBKG* mutations can mediate alternative mRNA splicing and increased expression of the NEMO-Δex5 isoform in patient cells, which is associated with activation of NF-κB response genes. In view of the clinical and molecular features that are distinct from patients with classical EDA-ID but shared with patients with other autoinflammatory diseases, we propose to name this disease NEMO deleted exon 5 autoinflammatory syndrome (NDAS).

### Defective TLR3- and RLR- but not TNF-induced NF-κB activation in dermal fibroblasts that express high levels of NEMO-Δex5.

**Figure 2:**
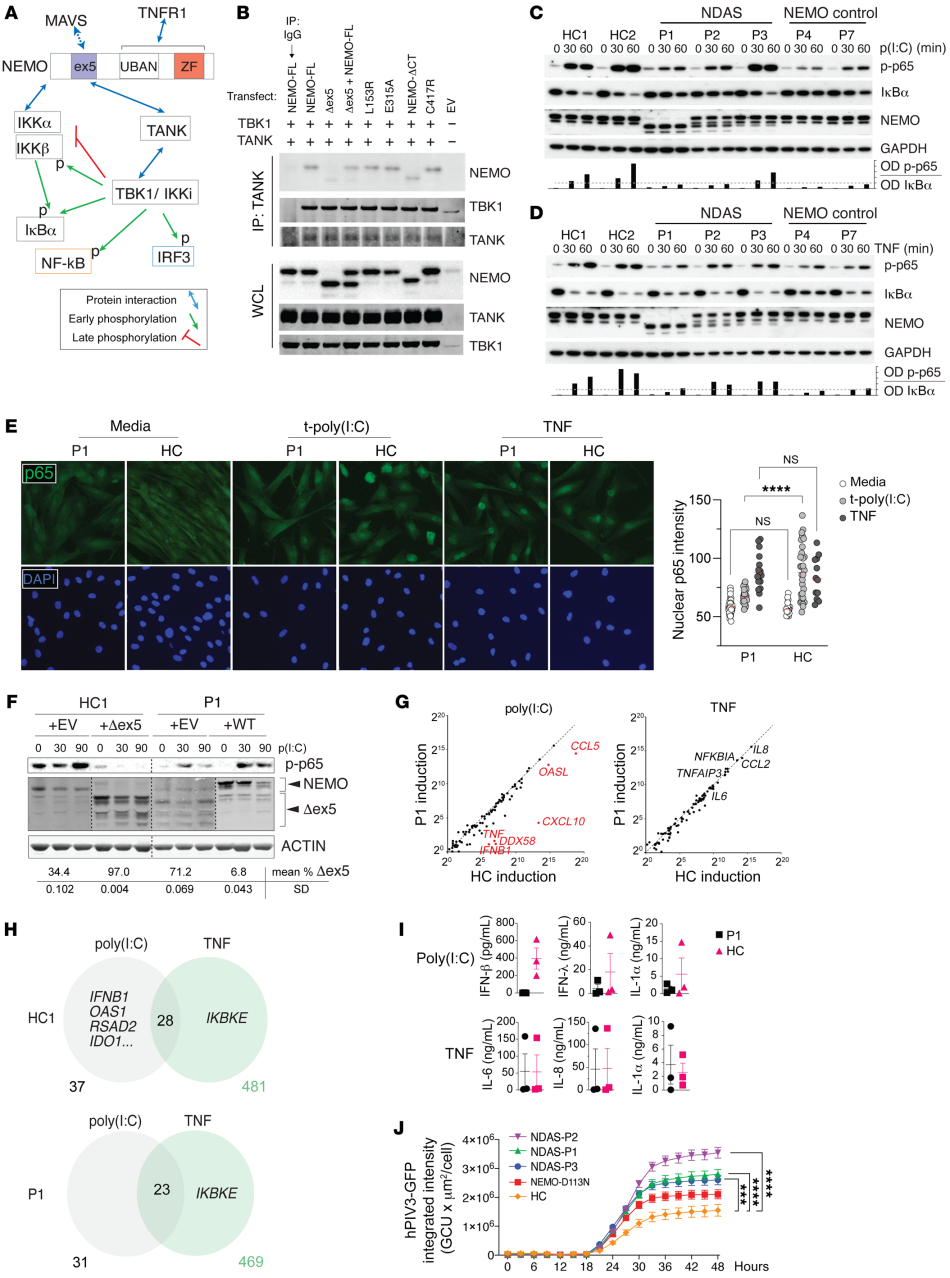
NEMO-Δex5 blocks NF-κB activation in response to poly(I:C) but TNF-induced NF-κB remains intact. (**A**) NEMO/TANK/TBK1 interaction and function. (**B**) HEK293T cells transfected with TANK, TBK1, and NEMO followed by TANK IP and Western blot as indicated. (**C** and **D**) Patient and healthy control (HC) dermal fibroblasts (DFs) were stimulated with poly(I:C) in a time course followed by Western blot as indicated. Quantitation of phospho-p65/IκBα by optical densitometry, below. Dashed line at 20%. (**E**) P1 and HC DFs were stimulated with TNF or poly(I:C) for 60 minutes and stained for NF-κB p65 visualized at 200× magnification by immunofluorescence (Keyence BZ9000), quantitation of p65 nuclear intensity, right. Statistical significance by 2-way ANOVA with Tukey’s multiple-comparison test. (**F**) DFs from HC and P1 were stably transduced with NEMO-Δex5 or FL-NEMO, respectively, and stimulated with poly(I:C) in a time course; lysates probed by Western blot as indicated. Different exposure intensity of NEMO panels (HC/EV, HC/Δex5, P1/EV, and P1/WT) was required to reveal all relevant bands, relative quantitation by optical densitometry, below. (**G**) P1 and HC DFs stimulated with poly(I:C), TNF, or cultured in media for 3 hours. Average gene expression by NanoString, ratio in stimulated/untreated from *n =* 3 independent experiments. (**H**) DFs treated as in **G** with relevant differentially expressed genes by RNA-Seq and totals shown, *n =* 3 technical replicates per media and TNF condition, *n =* 2 for poly(I:C) treatment. (**I**) P1 and HC DFs stimulated with TNF or poly(I:C) for 12 hours, cytokine production by capture bead assay, representative experiment of *n =* 3. (**J**) DFs from patients with NDAS, NEMO controls, and HCs incubated with hPIV3-GFP. Quantitation of total GFP intensity per cell, error bar indicates SEM. Two-way ANOVA applying Dunnett’s multiple-comparison test for adjusted *P* values, pertaining to 48-hour point. ***P < 0.001; ****P < 0.0001. Poly(I:C) dose 10 μg/mL and TNF 20 ng/mL throughout. All results representative of at least 2 independent experiments.

The domain encoded by exon 5 was suggested to mediate interaction of NEMO with TANK, which forms a ternary complex with TBK1 (Figure 2A and ref. 1). We therefore tested whether NEMO-Δex5 mutant might fail to associate with TBK-1 in cells transfected with NEMO-Δex5, FL-NEMO, or both. Indeed, NEMO-Δex5 pulldown with TANK and associated TBK1 was greatly reduced, even in cells cotransfected with FL-NEMO, in contrast to other previously described patient-derived NEMO mutations (Figure 2B and Supplemental Figure 2A). To investigate whether impaired NEMO-Δex5 association with TANK could affect TBK1 kinase activity, we tested IRF3 and NF-κB activation in response to poly(I:C), a ligand of both TLR3 and the cytosolic RLR family in patients’ dermal fibroblasts. Phosphorylation of IRF3 in response to poly(I:C) was intact (Supplemental Figure 2B). However, IκBα degradation and phosphorylation of the canonical p65 subunit of NF-κB was variably impaired in all 3 patients and correlated with levels of FL-NEMO expression such that the most impaired NF-κB activation was observed in cells from patient P1, whereas cells from patient P3 exhibited a response to poly(I:C) indistinguishable from healthy controls (Figure 2C). In response to the prototypical inflammatory cytokine TNF, NEMO-Δex5–expressing patient fibroblasts induced normal IκBα degradation and p65 phosphorylation (Figure 2D). Fibroblasts from P4 (expressing NEMO-C417R) demonstrated impaired NF-κB activation in response to both poly(I:C) and TNF (Figure 2, C and D). As an orthogonal measure of NF-κB activation, we measured nuclear translocation of NF-κB p65 after TNF and poly(I:C) stimulation. In P1 fibroblasts, nuclear translocation was normal in response to TNF but defective in response to transfected poly(I:C) (Figure 2E). We interrogated whether another TLR agonist, LPS, would activate NF-κB in fibroblasts from patients with NDAS compared with control fibroblasts. LPS stimulation led to modestly enhanced NF-κB translocation in fibroblasts from patients compared with healthy controls (Supplemental Figure 2C). To test whether the NEMO-Δex5 isoform could function to suppress signaling in the presence of FL-NEMO protein, we stably expressed the NEMO-Δex5 form in healthy control fibroblasts, which reduced poly(I:C)-induced NF-κB activation in healthy control cells. Conversely, expression of FL-NEMO rescued the NF-κB activation defect in P1 dermal fibroblasts (Figure 2F). These data suggest that the degree of poly(I:C)-induced NF-κB activation was affected by levels of NEMO-Δex5 and FL-NEMO.

To study the consequences of altered activation of NF-κB in fibroblasts expressing NEMO-Δex5, we measured the induction of a set of target genes using NanoString technology and RNA-Seq. TNF-induced gene expression in P1 skin fibroblasts was intact; however, poly(I:C) failed to induce *IFNB1*, *OAS1*, *RSAD2*, *IDO1*, and several other IFN-stimulated genes (Figure 2, G and H). In contrast, LPS stimulation led to equivalent expression of the majority of NF-κB response genes (Supplemental Figure 2D). Gene set enrichment analysis (GSEA) indicated enhanced TNF-mediated NF-κB signaling genes in response to LPS signaling in P1 cells compared with healthy control fibroblasts (Supplemental Figure 2E). To determine whether the NEMO-Δex5 isoform alone was able to propagate TNF- or poly(I:C)-induced signaling leading to NF-κB activation, we retrovirally expressed NEMO-Δex5 in NEMO-deficient cell lines. We were unable to expand primary human dermal fibroblasts from single-cell cultures after NEMO deletion and therefore generated a NEMO-deficient HEK293T cell line to study signal transduction mediated by either isoform in isolation. Whereas NEMO-deficient Jurkat T cells or HEK293T cells were unable to activate NF-κB in response to TNF, expression of either FL-NEMO or NEMO-Δex5 in these cells led to reconstitution of TNF-induced NF-κB pathway activation. In contrast, whereas expression of either NEMO isoform in knockout T cells led to NF-κB activation after poly(I:C) stimulation, NEMO-deficient HEK293T cells reconstituted with FL-NEMO responded to poly(I:C) stimulation with NF-κB activation, but cells expressing NEMO-Δex5 alone did not (Supplemental Figure 2, F and G). However, it is possible that dermal fibroblasts that exclusively express high levels of NEMO-Δex5 may not exhibit a similar pattern due to cell type–specific signaling differences.

Consistent with impaired induction of gene expression in response to poly(I:C) in patient fibroblasts, secretion of IFN-β, IFN-λ, and the proinflammatory cytokine IL-1α were reduced (Figure 2I). Conversely, TNF-induced production of the proinflammatory cytokines IL-6, IL-8, and IL-1α was intact (Figure 2I). To test the impact of altered signaling and cytokine production in the setting of viral infection, we infected patient skin fibroblasts with a GFP reporter strain of human parainfluenza virus 3 (hPIV3), a single-stranded RNA virus (19–22). Primary skin fibroblasts or induced pluripotent stem cell–derived (iPSC-derived) fibroblast-like cells from patients P1, P2, and P3 infected with hPIV3-GFP permitted increased viral protein expression compared with healthy control cells and other NEMO mutant cell lines (Figure 2J and Supplemental Figure 2H).

### NEMO-Δex5 mediates NF-κB activation in immune cells and excess type I IFN production.

The reduced antiviral responses in fibroblasts from patients with NDAS would suggest a more severe primary immunodeficiency than has been observed clinically in these patients. We hypothesized that the effects of NEMO mutations in NDAS may be tissue specific and evaluated the effects of the NEMO-Δex5 isoform in immune cells.

**Figure 3:**
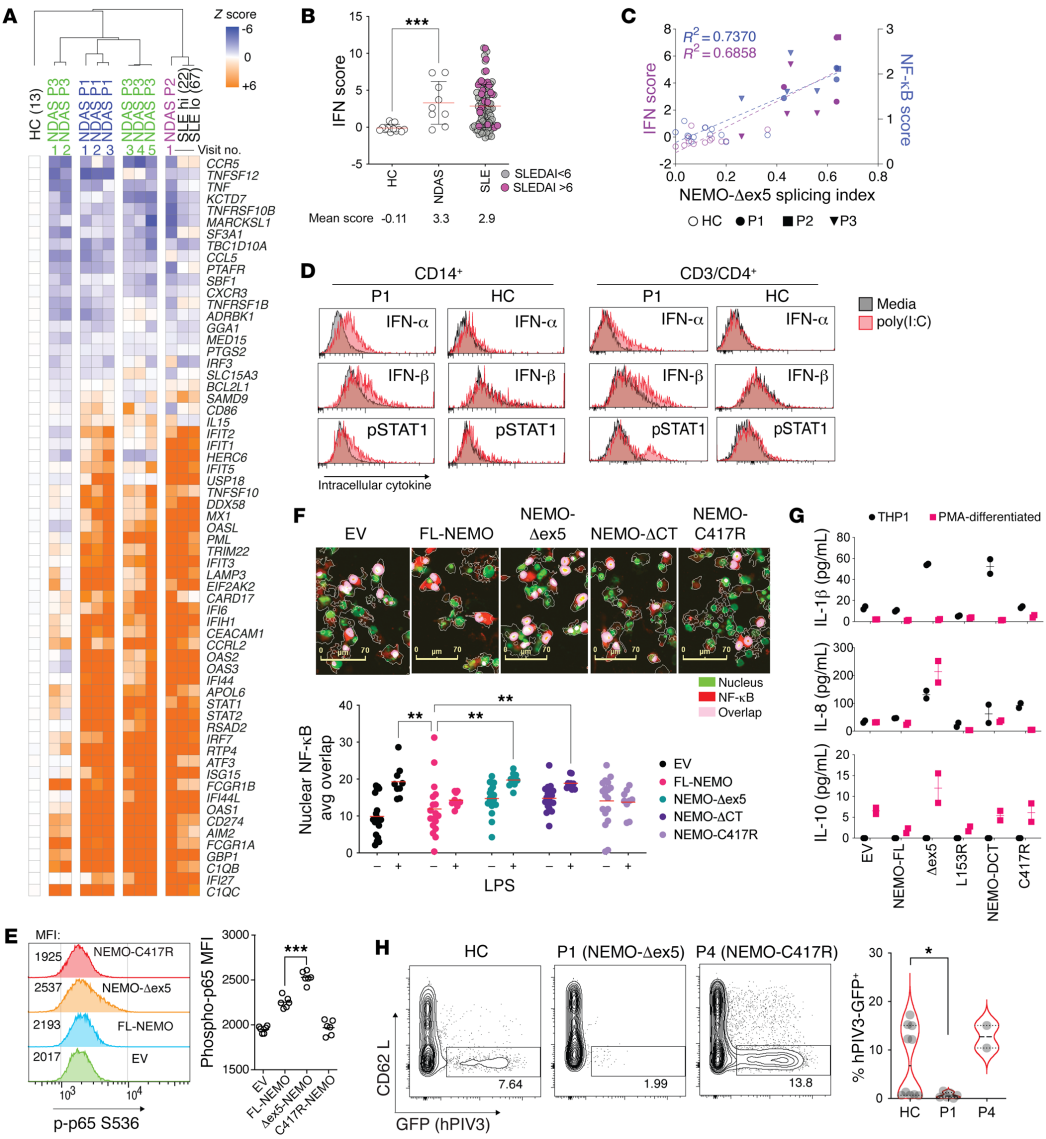
Increased NF-κB activation and type I IFN production in T cells and monocytes expressing NEMO-Δex5. (**A**) Relative gene expression by whole-blood RNA-Seq from P1, P2, and P3 obtained at serial clinic visits; patients with systemic lupus erythematosus (SLE) with SLEDAI greater than 6 (SLE hi, *n =* 22); SLEDAI less than 6 (SLE lo, *n =* 76), and from healthy controls (*n =* 13). (**B**) IFN signature gene score (see Methods) from whole-blood RNA-Seq data, ordinary 1-way ANOVA, Dunnett’s correction for multiple comparisons, group mean indicated by red bar with SD. (**C**) IFN and NF-κB scores plotted versus NEMO-Δex5 splicing index, least squares linear regression fit, R^2^ shown. (**D**) P1 and HC PBMCs cultured in media or stimulated with poly(I:C) 10 μg/mL for 60 minutes and treated with brefeldin for 4 hours, stained with antibodies against IFN-α, IFN-β, p-STAT1, CD14, and CD4. (**E**) THP1 were stably transduced to express FL or mutant forms of NEMO. NF-κB p65 phosphorylation was measured by intracellular flow cytometry. (**F**) PMA-differentiated reconstituted THP1 cells were stimulated with LPS 10 ng/mL for 60 minutes, fixed, and stained with anti–NF-κB p65 (red) and nuclear dye (green). Scale bars: 70 μm. Areas of nuclear p65 overlap (pink) were quantitated (below). Means compared by 2-way ANOVA with Dunnett’s correction. (**G**) Secreted cytokines in cell culture supernatant from NEMO-reconstituted THP1 cells (black symbols) after 72-hour PMA differentiation (red). (**H**) P1, P4, and HC blasting T cells were infected with GFP-expressing human parainfluenza virus 3 (hPIV3-GFP) for 48 hours; *n =* 6, 2, and 10 replicates, respectively. Frequency of hPIV3-GFP^+^ cells, right. *P* value calculated with Mann-Whitney unpaired, 2-tailed *U* test. All data except from Figures 3A–C are representative of at least 2 independent experiments. *P < 0.05; **P < 0.01; ***P < 0.001.

We serially collected independent whole-blood samples from patients P1–P3 and performed RNA-Seq. Analysis of gene expression profiles by Ingenuity Pathway Analysis (IPA) indicated differential induction of genes in 28 functional pathways, including “acute phase response signaling” and 3 pathways associated with IFN and antiviral signaling in patient P1 (Supplemental Figure 3A). Induction of these pathways was consistent with early observations of an IFN response gene signature in PBMCs from all 3 patients with NDAS. To quantify the expression of IFN response genes, we compared the IFN transcriptional signature in patients P1, P2, and P3 and patients with systemic lupus erythematosus (SLE), a known type I IFN–associated disease, with that of healthy controls (23). We examined expression of a set of 64 IFN-stimulated genes in peripheral blood from patients P1–P3 obtained throughout the course of their evaluation. The IFN-stimulated genes from patients P1 and P2 and 3 out of 5 samples from P3 were elevated and grossly similar to the expression in patients with SLE (Figure 3A). We next sought to determine whether the NDAS class of mutation exhibited gene expression patterns distinct from other previously described NEMO mutations. Because whole-blood samples from patients P4, P5, and P7 were not available, we performed RNA-Seq and gene expression analysis on unstimulated PBMCs previously isolated from these patients, which we compared with similarly isolated samples from patients P1, P2, and P3. GSEA indicated enhanced type I and type II IFN response in NDAS samples compared either with healthy controls or “other NEMO” (i.e., P4, P5, and P7) and conversely, downregulated TNF signaling in other NEMO relative to NDAS samples (Supplemental Figure 3B and Supplemental Table 1). To quantify increased expression of IFN-stimulated genes, we calculated an IFN score from whole blood, indicating that the mean IFN score of all 3 patients with NDAS was comparable to that of patients with SLE with a range of disease activity (Figure 3B, Supplemental Figure 3B, and Supplemental Table 1). Although samples from patients with NDAS exhibited a mean IFN score similar to that of patients with SLE as a whole, we noted variability in the expression of these genes, particularly for samples taken from patient P3 (Figure 3A). To determine whether the IFN score correlated with the extent of alternative exon 5 splicing, we plotted the IFN score against the percentage of alternate splicing of NEMO transcript obtained from patients with NDAS and 15 healthy controls (Figure 3C). This analysis indicated a significant correlation between the IFN score and alternative splicing (R^2^ = 0.69, *P <* 0.0001). A similarly high correlation with the NF-κB score was found (R^2^ = 0.74 *P <* 0.001; Figure 3C). Samples from patient P3 exhibited a normal IFN score when alternative splicing was low (IFN score = 0.08 with splicing index of 0.28). However, the IFN and NF-κB scores were among the highest of all patients with NDAS when the splicing index approached 50%. We found that alternative splicing of NEMO was also highly correlated to the “3-genes NF-κB/STAT1score” that distinguishes patients with NDAS from those with other type I interferonopathies, such as chronic atypical neutrophilic dermatosis with lipodystrophy and elevated temperature (CANDLE) and stimulator of IFN genes–associated (STING-associated) vasculopathy with onset in infancy (SAVI), and consistent with our NF-κB correlation, the “11-gene NF-κB-only validation score” was also highly correlated with alternative NEMO splicing (Supplemental Figure 3C and ref. 24). To directly measure IFN production in cells from patients with NDAS, we stimulated PBMCs from patient P1 with poly(I:C) and measured intracellular cytokine production by intracellular flow cytometry. P1 CD14^+^ cells and CD4^+^ T cells had enhanced type I IFN and STAT1 phosphorylation compared with cells from healthy controls, indicating that IFN induction and signaling pathways were intact (Figure 3D and Supplemental Figure 3, B, D, and E). These results indicate that in contrast to skin fibroblasts, peripheral blood CD14^+^ and CD4^+^ T cells expressing high levels of NEMO-Δex5 are responsive to poly(I:C) stimulation and produce an excess of type I IFN.

We generated T cell and monocyte cell lines that stably expressed FL-NEMO, the NEMO-Δex5 isoform, or previously described gain-of-function (E391X) or loss-of-function (i.e., C417R) mutants. Unlike the findings in fibroblasts, expression of NEMO-Δex5 in the undifferentiated THP-1 monocytic cell line led to increased constitutive and poly(I:C)-induced p65 phosphorylation compared with WT NEMO (Figure 3E and Supplemental Figure 3F). NEMO-Δex5–reconstituted cells differentiated with PMA were also more responsive to TLR stimulation than cells expressing FL-NEMO or the known loss-of-function mutant C417R (Figure 3F), and both undifferentiated and PMA-differentiated monocytic NEMO-Δex5 mutants secreted increased NF-κB response cytokines, such as IL-1β, IL-8, and IL-10 (Figure 3G). In Jurkat T cells, transfection with poly(I:C) to target RLRs yielded greater activation of NF-κB in NEMO-Δex5 cells compared with cells expressing FL-NEMO (Supplemental Figure 3G).

The intact or enhanced IFN response to poly(I:C) in immune cells harboring high levels of NEMO-Δex5 contrasts with findings in skin fibroblasts (Figure 2) and indicated that haematopoietically derived cells from patients with NDAS may not be similarly susceptible to viral infection in vitro. To test whether the observed enhanced IRF3 response and IFN-stimulated gene expression increased the ability of P1 immune cells to resist viral infection, we cultured primary blasting T cells with hPIV3. In comparison to healthy control cells, P4 (C417R loss of function NEMO) cells were hypersusceptible to hPIV3 infection, whereas P1 cells were less susceptible (Figure 3H).

### NEMO-Δex5 stabilizes IKKi to enhance NF-κB signaling in immune cells.

**Figure 4:**
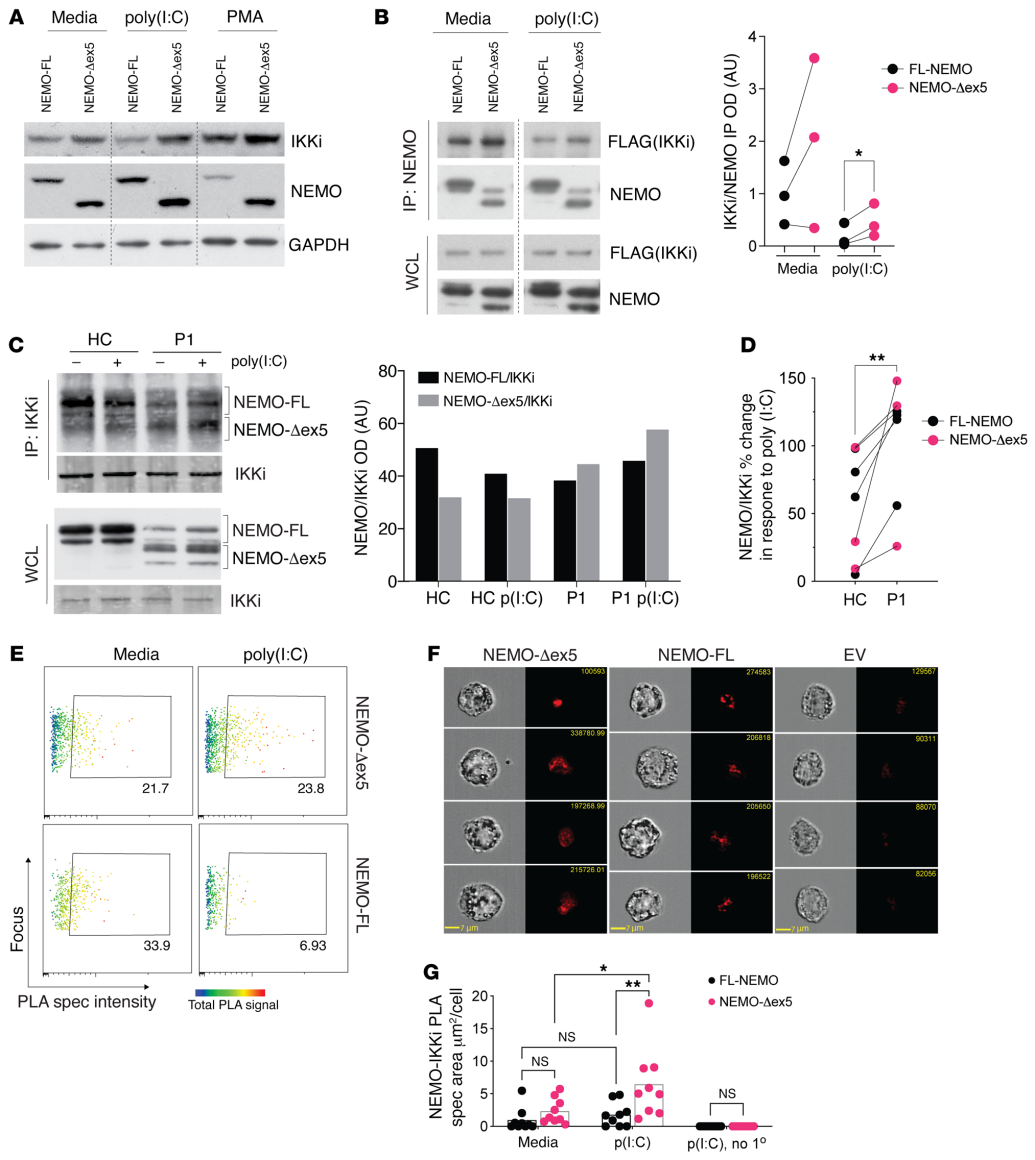
NEMO-Δex5 and IKKi form a stable complex in response to poly(I:C) stimulation. (**A**) THP1 cells reconstituted with FL and mutant NEMO forms and stimulated with poly(I:C) for 90 minutes or differentiated with PMA for 72 hours followed by Western blot of whole cell lysates. (**B**) HEK293T cells were transfected with FL or NEMO-Δex5 followed by stimulation with poly(I:C) 10 μg/mL for 8 hours and IP of IKKi using FLAG epitope. The membrane was probed for IKKi and NEMO with quantitation of pulled down IKKi normalized to NEMO on the right; means compared by paired, 2-tailed *t* test. (**C**) HC and P1 blasting T cells stimulated with poly(I:C) 10 μg/mL for 3 hours and IKKi isolated by IP. Western blot to detect co-immunoprecipitated NEMO, and IKKi blot as IP control, (right) quantitation of NEMO pulldown with IKKi by densitometry, black bars correspond to FL-NEMO bands and gray bars to NEMO-Δex5 forms in top panel (ImageJ). (**D**) Quantitation of total NEMO pulldown (NEMO-FL + NEMO-Δex5) as in **C**, shown as poly(I:C) response [ratio of poly(I:C) treated/media] (*n =* 4 independent IPs from NDAS-P1 and 4 IPs from HC T cells; means compared by paired, 2-tailed *t* test. Lines link paired data obtained from the same experiment. (**E**) NEMO-reconstituted THP1 cells in media or stimulated with LMW poly(I:C) 10 μg/mL for 60 minutes with NEMO and IKKi proximity ligation assay (PLA) spec intensity (*x* axis) and total NEMO/IKKi complex intensity (rainbow heatmap) using specific antibodies against IKKi and NEMO. (**F**) Four representative images taken at 40× original magnification of cells treated for 60 minutes as in **E**, with average signal area quantified; means compared by 2-way ANOVA with Tukey’s correction (**G**). EV, empty vector; FL, full length; no 1°, omission of primary antibody control. All results representative of at least 2 independent experiments. *P < 0.05; **P < 0.01.

We investigated potential molecular mechanisms underlying the proinflammatory cytokine and IFN production associated with NEMO-Δex5 expression. We considered the atypical IKK kinases TBK1 and IKKi because they are known to interact with NEMO and have roles in IFN induction (25–28). Whereas TBK1 is expressed constitutively in several cell types, IKKi has been shown to be induced by activators of NF-κB, such as LPS and PMA, and is constitutively expressed at higher levels in leukocytes compared with skin cells (28). We detected endogenous IKKi protein in THP-1 cells by Western blot that was further induced in response to PMA and to poly(I:C) (Figure 4A). To investigate the interaction between IKKi and NEMO isoforms, we reconstituted FL-NEMO or NEMO-Δex5 in NEMO-deficient HEK293T cells. NEMO-Δex5 readily associated with IKKi when both were expressed at high levels (Figure 4B). After stimulation with poly(I:C), IKKi association with FL-NEMO was reduced, even though total levels of transfected IKKi remained stable. In cells expressing NEMO-Δex5, IKKi association persisted in a complex with NEMO after poly(I:C) stimulation (Figure 4B, quantitation of *n =* 3 experiments)

To determine whether NEMO-Δex5 also associates with and stabilizes the NEMO-IKKi complex in primary T cells, we immunoprecipitated IKKi from T cell blasts from patient P1 and a healthy control with or without poly(I:C) stimulation and detected associated NEMO isoforms by Western blot. FL-NEMO was associated with IKKi in unstimulated healthy control cells (Figure 4C). After stimulation with poly(I:C), levels of IKKi-associated FL-NEMO were somewhat reduced (Figure 4C). In T cells from patient P1, however, poly(I:C) treatment did not lead to a reduction in IKKi-associated NEMO-Δex5 (Figure 4C). In addition, in contrast to cells from healthy controls, FL-NEMO association with IKKi was not reduced in P1 cells after poly(I:C) treatment (Figure 4, C and D). Given the high background signal resulting from the limited availability of primary cells from patients, we sought to quantitate the NEMO-IKKi stabilization by an independent method. The proximity ligation assay (PLA) has been used in single-cell measurement of heterotypic protein localization with a theoretical maximum distance of 30 to 40 nanometers (29, 30). Using 2 antibodies specific to NEMO and IKKi, we detected a NEMO-IKKi PLA association-signal in THP1 cells reconstituted with FL-NEMO and NEMO-Δex5. After stimulation with LMW poly (I:C), NEMO-IKKi complex intensity was reduced 4-fold in cells transduced with FL-NEMO, whereas cells transduced with NEMO-Δex5 maintained a stable NEMO-IKKi complex signal (Figure 4E). Individual cell image analysis revealed that the increased PLA signal in NEMO-Δex5 cells was due to larger NEMO/IKKi specs in these cells compared with those expressing FL-NEMO, suggesting oligomerization/aggregation (ref. 30 and Figure 4, F and G). Thus, NEMO-Δex5 can form a complex with IKKi that resists dissociation in response to poly(I:C) signals, which likely contributes to the enhanced NF-κB and IFN scores derived from cells expressing high levels of NEMO-Δex5 and IKKi.

### TNF induces IKKi expression and restores antiviral response in fibroblasts of patients with NDAS.

**Figure 5:**
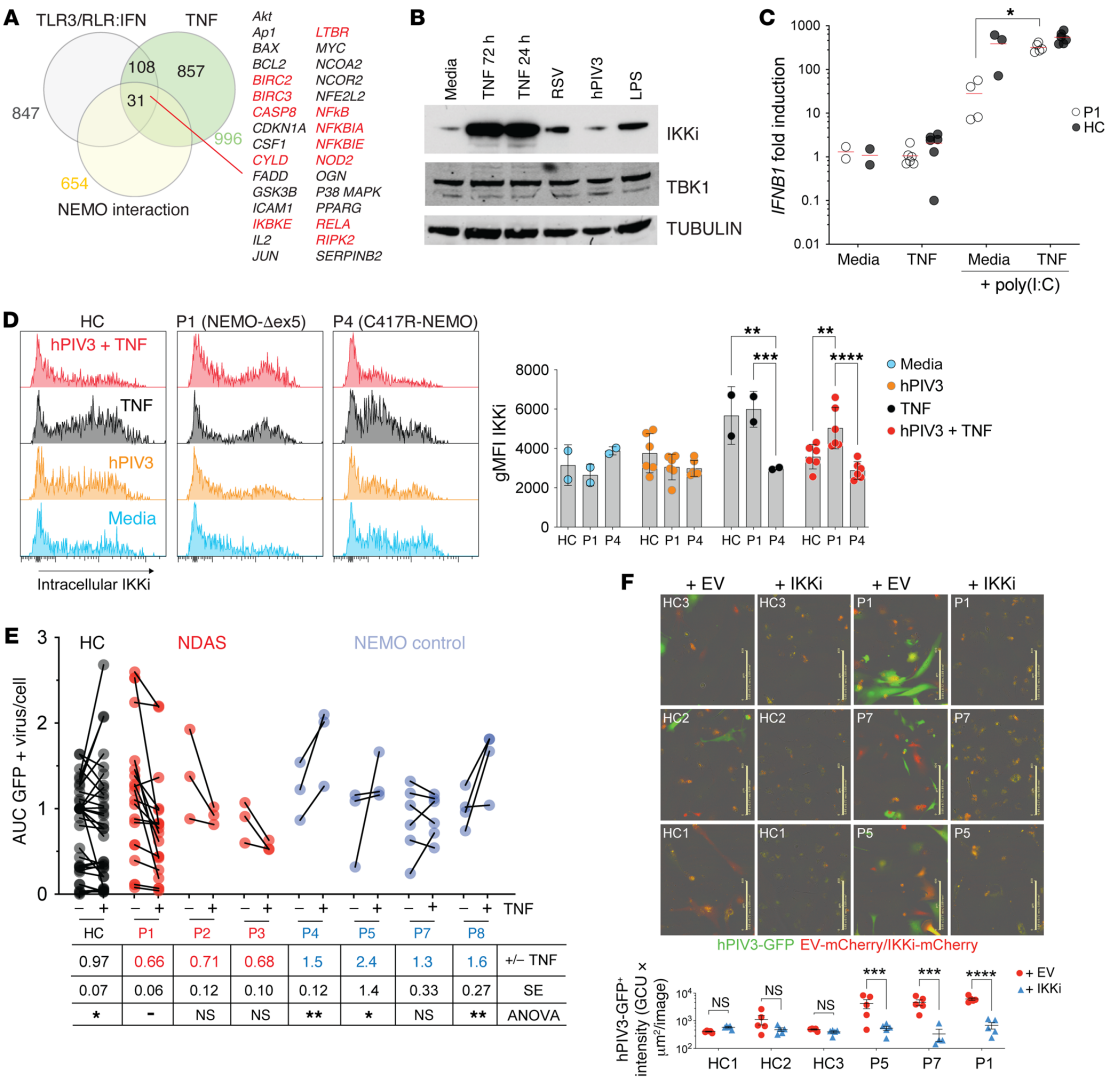
IKKi can be induced by TNF in hPIV3-infected P1 dermal fibroblasts, Ih rescues innate antiviral immunity. (**A**) Venn diagram indicates the intersection of TNF-inducible, TLR3/RLR/IRF3 pathway genes that interact with NEMO, both directly (red) and indirectly (black). (**B**) IKKi expression in primary dermal fibroblasts from HCs in response to TNF 20 ng/mL for 24 hours or TNF 20 ng/mL, LPS 10 ng/mL, or viral infection for 72 hours detected by Western blot. TBK1 and TUBULIN served as loading controls. (**C**) P1 and HC skin fibroblasts were stimulated with poly(I:C) 10 μg/mL for 3 hours with or without TNF 20 ng/mL costimulation, and *IFNB1* gene expression was determined by qPCR, 2-tailed Mann-Whitney *U* test. (**D**) P1, P4, and HC dermal fibroblasts were infected with hPIV3-GFP and treated with TNF 20 ng/mL as indicated by red shaded histograms. At 72 hours, cells were fixed and stained intracellularly with antibody to detect IKKi expression (quantitated on far right). (**E**) NDAS, NEMO control, and HC dermal fibroblasts (or iPSC-derived fibroblast-like cells from P5) were infected with hPIV3-GFP with or without TNF 20 ng/mL costimulation and imaged in time course experiments of 48 to 72 hours duration to measure virus protein expression. AUC of total GFP intensity per cell was measured and within-experiment normalization to HC samples was performed. For HC and NDAS-P1 samples, *n =* 7 independent experiments, for all other samples *n =* 3 independent experiments, with total numbers of conditions shown; “+/- TNF” in figure table underneath indicates mean hPIV3-GFP AUC ratio of TNF-stimulated versus unstimulated infected cells. Means compared by 2-way ANOVA with Tukey’s multiple-comparison test. (**F**) iPSC-derived fibroblast-like cells were transduced with lentiviral overexpression vector encoding IKKi-IRES-mCherry and infected with hPIV3-GFP. Scale bars: 400 μm. Two-way ANOVA with Bonferroni’s correction for multiple comparisons. Data are representative of at least 2 independent experiments. *P < 0.05; **P < 0.01; ***P < 0.001; ****P < 0.0001.

The observation of intact TNFR1 signaling in patient fibroblasts and ability of the NEMO-Δex5 isoform to stabilize the NEMO-IKKi complex and cause hyperresponsiveness to TLR-3/RLR stimulation in immune cells suggested a mechanism by which skin fibroblasts expressing NEMO-Δex5 could acquire enhanced antiviral activity. Using our RNA-Seq data (Figure 2H), we examined the subset of TNF- and LPS-induced genes known to be involved in the TLR3/RLR signaling pathway that interact directly or indirectly with NEMO (Figure 5A, see Methods). We identified *IKBKE*, encoding IKKi, which is activated by TNF and LPS stimulation in a NEMO-dependent manner. We confirmed that TNF and LPS treatment both robustly induced IKKi protein expression in dermal fibroblasts. In contrast, we noted only modest induction of IKKi protein after RSV infection, whereas hPIV3 infection alone did not appear to induce IKKi expression, perhaps because of a recognized virally mediated suppression mechanism (ref. 31 and Figure 5B). We tested whether TNF induction could rescue type-I IFN production in fibroblasts stimulated with poly(I:C) from patient P1. Indeed, TNF restored *IFNB1* expression in dermal fibroblasts treated with poly(I:C) from patient P1, although it had little effect on healthy control cells (Figure 5C).

We tested whether TNF might further enhance IKKi expression in P1 fibroblasts infected with hPIV-3. In contrast to IKKi induction following viral infection alone, infection of skin fibroblasts with hPIV3 in the presence of TNF led to a sustained increase in IKKi protein expression in P1 skin fibroblasts compared with fibroblasts from healthy controls and P4 (NEMO-C417R) (Figure 5D).

Because IKKi can substitute for TBK1 in the alternative IKK complex (32), we reasoned that expression of IKKi should restore the antiviral immune defect in NDAS dermal fibroblasts. TNF costimulation significantly reduced hPIV3 replication in cells from patients P1–P3 by approximately 30%. In contrast, TNF did not confer resistance to virus replication in P4 fibroblasts harboring the classic loss-of-function NEMO C417R mutant, any of the other control NEMO-mutant fibroblasts, or in cells from healthy controls (Figure 5E). To understand the specific role of IKKi induction in the suppression of viral replication, we retrovirally transduced patient and healthy control iPSC-derived fibroblast-like cells to overexpress IKKi (Supplemental Figure 4, A and B). Similar to NDAS patient–derived skin fibroblasts treated with TNF, IKKi-expressing cells from patient P1 demonstrated enhanced ability to resist hPIV3. Two other NEMO mutant controls also suppressed hPIV3 infection when IKKi was overexpressed, suggesting that their lack of TNF responsiveness was related to an inability to upregulate or properly signal in concert with endogenous IKKi (Figure 5F). These results suggest that inflammatory cytokines, such as TNF or LPS, can mitigate defective IFN production in NDAS skin fibroblasts expressing high levels of NEMO-Δex5, restoring their ability to control viral replication. In addition, IKKi-NEMO complex stabilization in cells that express high levels of NEMO-Δex5 presents a mechanism to understand the peripheral blood IFN and NF-κB gene signatures and autoinflammatory disease phenotype in these patients.

## Discussion

Here, we describe 3 male patients with a syndrome we term NDAS that arises due to germline mutations in splicing regulatory elements in *IKBKG* encoding NEMO. Patients with NDAS present with uveitis, a predominantly lymphohistiocytic panniculitis, hepatitis, and a striking lack of severe or recurrent infections, which distinguishes them from patients with other clinical syndromes linked to mutations in *IKBKG*. Patients with NDAS are characterized by increased IFN and NF-κB response gene signatures and a lack of cardinal features, such as basal ganglia calcifications, white matter disease, and hypertension seen in other type I interferonopathies (6, 33). Of additional clinical importance, standard exome filtering algorithms for missense mutations failed to detect all 3 NDAS patient mutations since they were deemed to be silent mutations. Our results thus highlight the need to consider mutations that may lead to alternative splicing and suggest that there may be additional patients with NDAS with mutations affecting *IKBKG* splicing that remain undiagnosed even after exome sequencing.

Consistent with previous studies of the NEMO-Δex5 isoform, (15, 34), we found that cells from patients with NDAS displayed unique NF-κB signaling features. Whereas NF-κB activation in response to TLR3/RLR stimulation was impaired in NDAS patients’ skin fibroblasts that expressed high levels of the NEMO-Δex5 isoform, it was intact in patients’ T cells and monocytes. Our results indicate that in contrast to TLR3 stimulation of unprimed skin fibroblasts, TLR3 stimulation of NDAS patient hematopoietic cells with poly(I:C) or by viral infection may lead to enhanced type I IFN production and antiviral responses due to a relatively stabilized NEMO-Δex5-IKKi complex. The ability of TNF-primed NDAS skin fibroblasts to stabilize IKKi levels and resist infection with hPIV3 (Figure 5, D–F) is consistent with a role for IKKi in NF-κB activation and innate antiviral immunity (25, 27, 28, 35). However, IKKi plays a secondary role in negatively regulating type I IFN induction, as bone marrow–derived macrophages (BMDMs) from IKKi knockout mice can produce increased IFN-β, potentially through RIG-I downregulation (4, 36). These apparently opposing functions for IKKi in NF-κB and type I IFN signaling have precedent: genetic knockout of TBK1, TANK, and TAK1 can mimic overexpression phenotypes in these signaling pathways (4, 37, 38). In any event, the negative regulatory functions of IKKi revealed by its deletion do not preclude a positive role for IKKi in TLR3/RLR-induced NF-κB and IFN induction and a critical role for IKKi in cells expressing NEMO-Δex5.

Systemic autoinflammatory disease (SAID) in patients with NDAS may result from, in part, enhanced stability of the NEMO-IKKi complex. Previous work, however, demonstrated impaired association of IKKi with NEMO-Δex5 (39). Various factors could explain this discrepancy. First, the IKKi-NEMO-Δex5 association may only be detectable when IKKi is expressed at high enough levels such that overexpression of the TANK adapter to bridge the association between NEMO and IKKi is not required. We showed that TNF and TLR ligands led to 10- to 15-fold upregulation of endogenous IKKi protein expression, and under these conditions, IKKi stabilization preferentially occurred in dermal fibroblasts from patients with NDAS (Figure 5, B and D). We also found that immune cells (i.e., activated T cells and THP1 monocytes) that constitutively express relatively high levels of endogenous IKKi contain a protein complex comprising IKKi, FL-NEMO, and NEMO-Δex5 (Figure 4, C–G). Therefore, one of the mechanisms leading to NEMO-Δex5-IKKi association may simply be increased IKKi protein levels. These transient, low-affinity interactions may be detectable only using optimized high-sensitivity techniques, such as PLA or immunoprecipitation in the presence of deubiquitinase inhibitors (40). A lower-affinity interaction mediated by polyubiquitin degradation or interactions would be consistent with stimulation-induced “tunable” and transient association, providing a mechanism for immune signaling regulation.

The stabilized IKKi-NEMO complex we observed in cells expressing high levels of NEMO-Δex5 might be due to impaired recruitment of ubiquitin modifiers that mediate IKKi-NEMO disassociation or degradation within the signaling complex. In murine alveolar macrophages, NEMO functionally interacts with a degradative E3 ligase, TRIM29, via residues that are absent in NEMO-Δex5 (41). A signaling complex in which FL-NEMO and NEMO-Δex5 are co-associated, therefore, would potentially lead to stabilization of FL-NEMO in cells that express NEMO-Δex5 after poly(I:C) treatment (Figure 4, C and D). Additionally, rather than failure to recruit an E3 ligase that may target IKKi, NEMO-Δex5 may fail to interact in vivo with negative regulators of IFN induction, such as LUBAC (37). Future work could be directed at identifying whether these or similar candidates are responsible for signaling abnormalities in cells that express high levels of NEMO-Δex5.

In classical NEMO hypomorphism or IKK-β deficiency, TNF leads to increased cell death, disrupting barrier integrity and triggering inflammatory response pathways that result in clinical features, such as colitis, that accompany immunodeficiency (42, 43). Other kindreds with severe immune deficiency and inflammatory disease have been described with a combination of branch-point mutation and stop-gain mutation in exon 5 (10, 14, 16). In these previous cases, NEMO-Δex5 protein was detected at low levels with only trace amounts of FL protein expression in patient cells. As a result, these cells were unable to activate NF-κB in response to TNF stimulation, in distinction to NDAS patient–derived cells, which, although exhibiting somewhat less than fully intact p65 phosphorylation, activated p65 sufficiently to permit the TNF-induced NF-κB translocation, gene expression, and protein upregulation of IKKi shown.

Consequently, we did not observe increased cell death after TNF stimulation of cells from patients with NDAS, and infection or colitis were not major clinical features. Our results suggest that sufficient expression of FL-NEMO may be required to develop an NDAS clinical phenotype distinct from the inflammatory disease due to IKK-β or NEMO hypomorphism in addition to other branch-point mutations that impair TNF signaling. TNF blockade has been a relatively effective treatment for 2 patients with NDAS (P1 and P3; ref. 24). Because of the potentiating effect of TNF on IKKi induction and the specific stabilization of IKKi by NEMO-Δex5, our results provide a mechanism by which TNF blockade may reduce excess NF-κB activity and inflammatory disease in patients with NDAS. Alternatively, the enhanced IFN gene expression signature found in NDAS suggests that blocking IFN receptor signaling may be beneficial. In contrast to other NEMO mutants, the NEMO-Δex5 isoform possesses an intact N-terminal domain, UBAN, and ZF domains that would permit association with IKK-α/β, K63-, and M1-linked polyubiquitin, respectively, which may explain its ability to activate NF-κB in response to TNF and LPS (44, 45). Consequently, NEMO-Δex5 differs from other NEMO mutants with impaired TNF-induced NF-κB activation or that fail to stabilize the NEMO-IKKi complex (Supplemental Figure 3B, Supplemental Figure 5, D and E, and ref. 5). Constitutively intact NF-κB function in NDAS patient hematopoietic cells or in skin fibroblasts after LPS or TNF signaling may account for the unique phenotype of systemic inflammatory disease without apparent increased susceptibility to viral or bacterial infection found clinically. Altered NF-κB signaling during development can lead to mild features of ectodermal dysplasia, such as conical teeth, which are likely due to impaired ectodysplasin A receptor (EDAR) signaling in these patients (46). The CNS involvement in NDAS differs from genetic TLR or RLR deficiencies leading to developmental disorders affecting bone and brain (47–51) in that cognitive development was grossly intact in patients with NDAS, even though CNS venous thrombosis occurred in 2 of 3 patients. As more patients are identified and systematically evaluated, a more complete understanding of the phenotypic spectrum in NDAS will likely emerge.

The mutations found in patients P1 and P2 lead to the expression of reduced levels of FL-NEMO in skin fibroblasts and failure to fully activate NF-κB in response to poly(I:C). Impaired poly(I:C)-induced NF-κB signaling coupled with intact TNF signaling was a pattern observed in fibroblasts from patients P1 and P2 and from NEMO patient P7 harboring NEMO E315A within the UBAN ubiquitin recognition domain that is involved in TLR3- and RLR-mediated NF-κB activation and antiviral responses (Figure 2, C and D and refs. 2, 52, 53). The intermediate responsiveness of P7 cells to TNF costimulation after hPIV3 infection (Figure 5E) suggests that TNF-mediated antiviral effects in NDAS cells rely on a unique combination of NF-κB activation that leads to induced IKKi expression coupled with IKKi stabilization that is specifically mediated by the NEMO-Δex5 isoform. Since the defect in poly(I:C)-induced NF-κB activation in P1 cells could be rescued by overexpression of FL-NEMO (Figure 2F), we would expect that P2 cells with higher levels of FL-NEMO and lower levels of NEMO-Δex5 expression would also be rescued by increasing expression of FL-NEMO. The ability to suppress normal signaling or rescue impaired signaling by overexpression suggests that variation in the ratio of FL-NEMO to NEMO-Δex5 isoforms or altering absolute levels of these isoforms regulates NF-κB activation.

After poly(I:C) stimulation of NDAS dermal fibroblasts, we observed phosphorylation of IRF3 at Ser386, although gene expression and cytokine production were impaired. Cells lacking NEMO or those reconstituted with NEMO-Δex5 alone fail to induce IRF3 dimerization and type I and type III IFN production in response to poly(I:C) (5, 39). Therefore, it appears that expression levels of FL-NEMO in NDAS cells are sufficient to enable poly(I:C)-induced IRF3 phosphorylation, but not NF-κB activation in response to poly(I:C), which is consistent with impaired IFN-β induction and diminished resistance to RNA virus replication in cells (54). Full IRF3 activation, like NF-κB activation, requires additional stimulation-induced posttranslational modifications that enable DNA binding, interaction with other transcription machinery, and normal gene expression. Given the cross-phosphorylation that occurs between the canonical IKK and the atypical IKK kinases and their substrates, it is possible that IRF3 posttranslational modifications other than S386 are dysregulated (55).

This raises the important question of how much NEMO-Δex5 isoform expression is required to develop a clinical phenotype. Although this cannot be determined from the present study, our results suggest that even relatively low expression of NEMO-Δex5 together with a low or normal expression of FL-NEMO may be sufficient. We note that “low” expression of NEMO-Δex5 in P3 skin fibroblasts is low only in comparison to the amount of FL-NEMO expressed. However, NEMO-Δex5 levels in NDAS patient cells was substantially more than that expressed by healthy control cells. Patient P3’s clinical phenotype seemed as severe as that of patients P1 and P2, although the skin fibroblast NEMO-Δex5/FL-NEMO ratio was lower and peripheral blood alternative splicing was on some occasions within the normal range (Supplemental Figure 1J). One possible explanation is that variable expression of NEMO-Δex5 in peripheral blood with transcript levels that can approach the normal range at times may be sufficient for pathological peripheral immune cell activation and gene expression resulting in a clinical disease phenotype.

Given the limitations in sample availability, we were unable to perform all experiments using cells from patients P2 and P3. Despite the strong association between the peripheral blood gene signature and disease phenotype, it is possible that some of the findings from P1 cells, such as reduced susceptibility to viral infection (Figure 3H), cytokine production (Figure 3D), and IKKi stabilization (Figure 4C and Figure 5D) do not occur in cells with lower levels of alternative splicing. Although we cannot be certain that the degree of primary immune deficiency (PID) or inflammation correlates with amount of FL-NEMO or NEMO-Δex5 isoform expressed, as more patients with NDAS are discovered and systematically studied, we hope to gain a deeper understanding of how relative levels affect the various possible roles and functions of this protein. We anticipate that future studies using compounds that modulate isoform expression in small animal models will enable a determination of the lowest amount of NEMO-Δex5 expression that leads to disease and altered immune function. Additionally, as the NEMO-Δex5 isoform is expressed in cells without *IKBKG* mutation, our results raise the possibility that monitoring and controlling exon 5 mRNA splicing may regulate innate immunity to pathogens and autoinflammatory disease in humans.

## Methods

### Primary cell isolation, sequencing, and cell stimulation.

Cultured dermal fibroblasts were obtained from collagenase type IV treatment of forearm skin biopsies. PBMCs were isolated from whole blood using Ficoll-Paque (Amersham Biosciences) gradient centrifugation. T cell blasts were generated by stimulation of total PBMCs with Concanavalin-A (Sigma-Aldrich) for 48 hours and expanded in IL-2 (NCI Preclinical Repository). PBMC subsets were isolated through positive selection using the AutoMACS Pro (Miltenyi Biotec) and anti-CD14 microbeads, anti-CD4–phycoerytherin (anti-CD4–PE) with anti-PE microbeads, and anti-CD8 FITC with anti-FITC microbeads (Miltenyi Biotec). For sequencing preparation, total RNA was harvested from primary cells using the RNeasy Mini Kit (Qiagen) and cDNA was synthesized using the SuperScript III First-Strand Synthesis SuperMix for qRT-PCR (Invitrogen). Sanger sequencing of cDNA was performed using appropriate primers. For cell stimulations, the following reagents were utilized: poly(I:C) (Sigma-Aldrich), low molecular weight (LMW) poly(I:C) (InvivoGen), Lipofectamine 2000 (Life Technologies), and recombinant human TNF-α (R&D Systems). Skin fibroblasts were stimulated with 10 μg/mL poly(I:C) alone [poly(I:C)] or along with transfection reagent, Lipofectamine [t-poly(I:C)]. TNF dose used for stimulation was 20 ng/mL unless otherwise specified.

### Viral infection assay.

Adherent cells were infected with hPIV3-GFP and viral protein expression was measured by acquiring serial images using a brightfield and green filters for GFP. AUC of total GFP intensity per cell was measured and within-experiment normalization to healthy control samples was performed.

### Cell lines.

NEMO-WT and NEMO-Δex5 T cell lines were generated through reconstitution of the NEMO-deficient Jurkat line 8321, provided by A. Ting (Mt. Sinai Hospital, New York, NY). Stable reconstitution of Jurkat and THP1-dual cells (InvivoGen) was performed using the Phoenix helper-free retrovirus producer lines as described previously (12). Phoenix cells were transfected with Migr1 plasmids containing FL-NEMO or NEMO-Δ ex5 cDNA sequences and an internal ribosomal entry site (IRES) GFP sequence. Two days later, virus was harvested and used to transduce Jurkat line 8321. GFP^+^ NEMO-reconstituted cells were selected by FACS. For transient transfection of HEK293T cells (ATCC), X-tremeGENE HP DNA Transfection reagent (Roche) was utilized to deliver Migr1 plasmids containing FL or NEMO-Δex5 cDNA sequence. Biochemical experiments were performed 48 hours after transfection. iPSC-derived fibroblast-like lines were generated using episomal expression plasmids to generate iPSCs and then cultured in SB/E6 media (12).

### IKKi shRNA knockdown and overexpression.

Patient and healthy control iPSC-derived fibroblast-like cells were lentivirally transduced. IKKi gene was subcloned into a lentiviral vector, pLVX-IRES-mCherry (Takara), and transfected into Lenti-X HEK293T cells (Takara) with Lenti-X Packaging Single Shots (Takara). Two days later, supernatants were collected and passed through a 0.22 μM filter. Lentivirus titer was measured with Lenti-X GoStix kit (Takara).

### p65 staining in primary dermal fibroblasts and PMA-differentiated THP1 cells.

Twenty-four hours prior to stimulation, primary fibroblasts were trypsinized and plated onto Millicell EZ slides (MilliporeSigma). After stimulation, cells were fixed with paraformaldehyde and permeabilized in saponin (0.1%). Blocking was performed with 5% goat serum and stained with anti-p65 (1:100, Santa Cruz Biotechnology, sc-372) overnight and Alexa Fluor 488–conjugated goat anti-rabbit IgG (1:1000, Invitrogen, A27034) for 1 hour prior to mounting in ProLong Gold with DAPI (Life Technologies) overnight. Cells were then imaged using the BZ-9000 series Biorevo 20× objective (Keyence). Quantitation of nuclear p65 intensity was performed using ImageJ without normalization to total p65 (56).

NEMO-reconstituted THP1 cells were treated with 2 μM PMA (MP Biomedicals) for 72 hours. Media was aspirated and incubated in PBS with or without 10 ng/mL LPS for indicated times. Cells were fixed with 2% paraformaldehyde solution, permeabilized with perm/wash 1 buffer (BD Biosciences, 557885), and stained with anti-p65 (1:100, Santa Cruz Biotechnology, sc-372) and Alexa Fluor 568–conjugated goat anti-rabbit IgG (1:1000, Invitrogen, A11011). Reconstituted cells were identified by GFP signal. Nuclei were stained with Syto21 (1:1000, Thermo Fisher Scientific, S7556). Cells were imaged with IncuCyte S3 10× objective (Essen Bioscience) and coincident object analysis using integrated software.

### CBA assay in undifferentiated/PMA-differentiated THP1 cells.

NEMO-reconstituted THP1 cells were treated with 10 ng/mL PMA for 72 hours, and then rested for 24 hours in PMA-free complete D10 media. Cells were treated with 10 μg/mL HMW poly(I:C) for 72 hours, and then the supernatants were collected. Undifferentiated THP1 cells were rested for 3 hours in complete R10 and treated with 10 μg/mL HMW poly(I:C) for 20 hours, at which point supernatants were collected. Cytokines were measured from the supernatants using the Cytometric Bead Array (CBA) Human Inflammatory Cytokines kit (BD Biosciences, 551811), following the manufacturer’s protocol.

### PLA/PLIC.

NEMO-reconstituted THP1 cells were treated with HMW poly(I:C) or carrier. The samples were fixed as above and stained with anti-IKKi (1:100, Cell Signaling Technology, 3416) and anti-NEMO (1:200, BD Biosciences, 611306) blocked with 5% donkey serum and incubated with donkey anti-rabbit plus and anti-mouse minus probes (Sigma-Aldrich, DUO92002, DUO92004) in situ detection reagent red (Sigma-Aldrich, DUO92008) following the manufacturer’s protocol. Images were acquired with multispectral imaging flow cytometry (40× and 60× objectives, ImageStream X Mark II flow cytometer, Amnis/EMD-Millipore), compensated, and processed using IDEAS software (Amnis). In focus, single cells were gated using area, aspect ratio, and gradient root mean squared, channel 01 (RMS Ch01). Stained cells were gated to exclude false positive signal (30); PLA^+^ specs were identified using R3 Bright Detail Intensity, corresponding to a 3-pixel signal radius. For area analysis, the 20× objective of an IncuCyte S3 (Essen) was used to acquire images, and analysis was done with IncuCyte software.

### Minigene system.

For minigene construction, a segment of genomic DNA spanning *IKBKG* exon 3 to exon 7 was amplified from patient- and healthy control–derived fibroblasts using Phusion High-Fidelity DNA Polymerase (New England Biolabs). Blunt-ended PCR products were cloned into pCR-Blunt vector (Invitrogen), subcloned into pCMV6-Entry vector (OriGene), and sequence-verified for use as minigenes. These vectors were sequence-verified to contain WT or patient *IKBKG* sequence. Minigene vectors were transfected into HEK293T cells using X-tremeGENE DNA Transfection reagent (Roche), and RNA was harvested 12 hours later using the RNeasy Mini Kit (Qiagen). After cDNA synthesis using the SuperScript III First-Strand Synthesis SuperMix for qRT-PCR (Invitrogen), PCR was performed using appropriate primers to selectively amplify the exogenous NEMO transcript from the minigene.

### IPA analysis.

A TLR3/RLR/IRF3 gene list was generated by selecting all 3 terms, using the “grow” function, and limiting terms to “experimentally observed.” Filtering for TLR3/RLR/IRF3 was limited to “expression” and “transcription” and for NEMO interaction to “protein:protein.” The IPA definition of “direct interaction” was used, which encompasses physical association and chemical changes, such as phosphorylation, dephosphorylation, ubiquitination, or degradation. Terms returned that corresponded to chemical compounds were excluded.

### Whole-genome and whole-exome sequencing variant analysis.

Gene variants detected in 185 known PID- and SAID-associated genes from patients P1–P3 were filtered by allele frequency in dbsnp and exac (<0.01), PolyPhen-2, Provean, and SIFT prediction and assuming X-linked, autosomal dominant de novo, or autosomal recessive inheritance.

### Healthy cohort allele frequency.

Whole-genome sequence profiles of a healthy adult cohort with no record of genetic disease (*n =* 1197) were used to assess reference base frequency at the 3 mutation sites observed in affected patients. Blood-derived genomic DNA samples from subjects were used as input for TruSeq PCR-free library preparation before whole-genome sequencing on Illumina HiSeq X platforms by single library, single-lane topography. The healthy cohort was composed of 1158 individuals of European descent, 8 individuals of African descent, 17 individuals of east Asian descent, and 14 Individuals of admixed American descent as defined by the 1000 Genomes super population classifications.

Given the genomic complexity of the *IKBKG* gene and its high degree of similarity to the adjacent region and *IKBKGP1* pseudogene, read-mapping quality scores were unreliable for alignment assessment. As a result, standard pipeline single nucleotide variant (SNV) calling was unavailable to provide an accurate representation for *IKBKG* variation distinct from *IKBKGP1*. To better understand the variation within a healthy cohort compared with affected patients, we measured base identity aligning to each locus within an individual without filtering for mapping quality of the read. Any read overlapping the *IKBKG* variant site and the homologous *IKBKGP1* was included, based on the observation that any read may equally align to the 2 sites due to the pair’s total sequence identity. Individual base calls with a PHRED score below 30 were excluded from the counts. Only individuals with at least 20 total reads overlapping the loci were included in the analysis.

### Co-immunoprecipitation and Western blot.

Cells were lysed in 20 mM Tris-HCl, 150 mM NaCl, 5 mM MgCl_2_, 1% (w/v) Triton X-100, 250 mM β-glycerophosphate, 10 mM sodium orthovanadate, 50 mM sodium pyrophosphate, 500 mM NaF, 10 mM sodium molybdate, 20 mM EGTA, 5 mM N-ethyl maleimide, 5 mM iodoacetamide, and cOmplete EDTA-free protease inhibitor (Roche). Insoluble cellular material was removed by centrifugation and immunoprecipitation was performed with appropriate antibodies and Dynabeads Protein G (Life Technologies). Western immunoblot was performed as previously described (56) using the following antibodies: p-Ser536-p65 (Cell Signaling Technology, 3033); NEMO (Santa Cruz, sc-8330; BD Biosciences, 611306); p65 (Santa Cruz, sc-372); IκBα (Santa Cruz, sc-371); p-Ser32/36-IκBα (Cell Signaling Technology, 9246); ACTIN (Abcam, ab3280); B-tubulin (Abcam, ab6046); TBK1 (Cell Signaling Technology, 3013); TANK (Cell Signaling Technology, 2141); p-Ser386-IRF3 (Abcam, ab76493); p-Ser396-IRF3 (Cell Signaling Technology, 4947).

### NEMO exon 5 splicing analysis.

The percentage of alternate splicing (%AS) of NEMO transcripts derived by RNA-Seq was defined as the ratio of mapped transcripts overlapping both exons 4 and 6 (4|6) to those overlapping both exons 4 and 5 (4|5), such that %AS = [(count 4|6) /(count 4|5)]. Indexed BAM files were visualized using Integrative Genomics Viewer (Broad Institute). For analysis of the predicted effect of mutations on splicing, the relevant sequence was uploaded to Human Splicing Finder version 3.1 (http://www.umd.be/HSF3/; ref. 57).

### Gene expression.

PAXgene tubes were used to collect RNA from patient and control whole blood. Total RNA was extracted using the RNeasy Mini Kit (Qiagen). Real-time quantitative PCR was performed on the CFX96 Real-Time PCR Detection System (Bio-Rad) using the iScript One-Step RT-PCR Kit for Probes (Bio-Rad) with appropriate primers and probes (Life Technologies). Multiplex quantitative gene expression measurements were obtained using the nCounter Analysis System (NanoString), utilizing a custom design CodeSet. For RNA-Seq, first- and second-strand cDNA was synthesized using the TruSeq RNA Sample Preparation Kit (Illumina) and sequenced using the HiSeq 2000 (Illumina). FastQ files were generated using CASAVA 1.8.2 and mapped to hg19 using Tophat 2.1.0. Hemoglobin and intergenic reads were removed using bedtools intersectBed. Reads per kb of transcript per million mapped reads (RPKM) values were calculated using Partek GS 6.6. Differential gene expression was conducted using DESeq2 in R using raw count data, the outputs of which include the FDR-adjusted *P* value of differential expression between a pair of conditions, as well as fold change for each gene. RPKM values were transformed to *Z* scores by subtracting the mean and dividing by the standard deviation of a set of healthy controls (*n =* 15). *Z* scores were subjected to hierarchical clustering and plotting of heatmaps using Morpheus (Broad Institute, MIT). The IFN response gene list was based on expression differences between CANDLE and SAVI patient samples versus NOMID and healthy controls, selecting genes based on IPA classification of IFN-regulated genes. The NF-κB response gene list was derived from intersecting the Gilmore Lab (Biology Department, Boston University, Boston, Massachusetts, USA) NF-κB target gene list and IPA experimentally observed results in humans.

### Cytokine measurement.

After stimulation of fibroblasts, culture supernatants were cleared of cellular debris by centrifugation. The concentrations of type I IFN and cytokines in the supernatants were assayed using the VeriPlex Human Cytokine 16-Plex ELISA Kit (PBL Assay Science) and the ChemiDoc MP Imaging System (Bio-Rad). For cytokine measurements from serum and PBMC supernatants, the Bio-Plex Pro Human Cytokine 27-plex assay (Bio-Rad) was utilized. For intracellular flow cytometry staining, IFN-α (Miltenyi Biotec, 130092602), IFN-β (PBL Assay Science, IFN 21400-3), IFN-γ (BD Biosciences, 554701), and p-STAT1 (BD Biosciences, 560310).

### Statistics.

Grouped experimental data are represented with mean ± SEM or when appropriate SD, as indicated in figure legends. Additional relevant statistical methods are described in the corresponding figure legends. Statistical analyses and graphing were done using GraphPad Prism, version 9. Outlier identification was performed using the robust regression and outlier removal (ROUT) method with *Q* = 0.5. Statistical comparisons between 2 groups were performed using 2-tailed Student’s *t* test unless otherwise stated. Other statistical analyses were performed using 1-way or 2-way ANOVA with repeated measures and Tukey’s, Dunnett’s, or Bonferroni’s corrections for multiple-comparison testing. A *P* value of 0.05 or less was considered to indicate a significant difference between groups. For GSEA, the FDR was set to 0.05.

### Study approval and clinical trial details.

The experimental protocol was approved by the IRB for Human Research at the NIH and Indiana University School of Medicine. For all patients and healthy controls enrolled, written informed consent was obtained in accordance with an NIH IRB-approved protocol. Patients were enrolled in trials NCT00001788, NCT00001372, and NCT02974595 (ClinicalTrials.gov).

## Author contributions

AWW, YL, JX, JGR, EL, SMK, APH, JZR, CE, AADJ, DNH, DGPP, BL, and EPH conducted experiments for this article. AWW, YL, JX, JGR, EL, SMK, APH, JZR, CE, SRB, ZD, AADJ, DNH, DGPP, CLD, SC, and EPH analyzed the data. AWW, YL, JX, JGR, EL, SMK, APH, JZR, CE, and EPH designed experiments. AWW, YL, JX, JGR, JZR, CE, AADJ, DNH, DGPP, MTT, VRD, CLD, SMH, RGM, RMS, and EPH helped with manuscript writing and revision.

## Supplementary Material

Supplemental data

## Figures and Tables

**Table 1:**
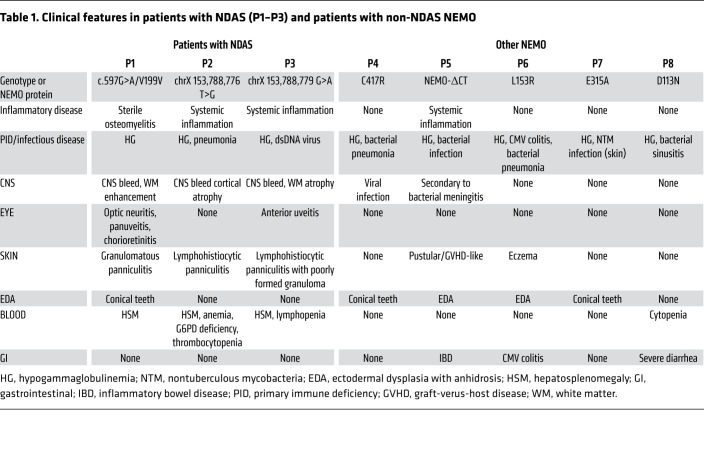
Clinical features in patients with NDAS (P1–P3) and patients with non-NDAS NEMO

## Version 1 03/15/2022

electronic version
