# Supplementary material for: Genetically programmed alternative splicing of NEMO mediates an autoinflammatory disease phenotype
Source: J Clin Invest. 2022 Mar 15;132(6):e128808. doi: 10.1172/JCI128808 (PMC8920334; doi:10.1172/JCI128808)
Supplement: Supplemental data [file jci-132-128808-s148.pdf]

Supplemental data:

Clinical Presentation and History

P1 (NEMO-∆ex5)

P1 is a Caucasian male of Iberian peninsula and Italian descent who was born following normal gestation that was complicated by late trimester primary maternal infection with Epstein Barr Virus and Cytomegalovirus. Birth parameters were normal, and 10 days following BCG inoculation at the age of 6 weeks, the patient developed fevers, secondary lymphoid organ hyperplasia, hepatosplenomegaly, diffuse panniculitis, generalized lymphadenopathy and leukocytosis with elevated markers of systemic inflammation. Comprehensive PCR to detect EBV, CMV, TORCH infections and bacterial, fungal and mycobacterial blood, lymph node, and CSF cultures were negative. Furthermore, special stains for mycobacteria were negative and no lesion at or near the site of BCG inoculation was found. Negative cultures and clinical response to pulse intravenous glucocorticoids followed by medium dose daily prednisone supported a diagnosis of auto-inflammatory disease. Skin and liver biopsy revealed a mixed inflammatory cell infiltrate with the presence of granuloma that did not contain bacilli by acid-fast stain and PCR. Axillary and right paratracheal lymph nodes, spleen, and celiac and retroperitoneal lymph nodes demonstrated increased metabolic activity by PET Scan. Soluble IL-2R level was increased at 19,584 U/ML [334-3026]. Lymph node biopsies at 14 months revealed preserved lymphoid architecture with numerous small noncaseating granulomas, some of which contained Langhans type giant cells. Special stains for fungi and mycobacteria of a lymph node were again negative as were stains for organisms of bone marrow biopsy. FDG PET scan at 14 months revealed abnormal uptake in the adenoid and salivary glands with enlarged cervical and supraclavicular lymph nodes and abdominal x-ray revealed marked hepatosplenomegaly with rightward shift of the stomach due to spleen enlargement (Figure S1C). Although the patient sweats normally and hair distribution and morphology are normal, eruption of primary dentition revealed conical incisors; therefore, a diagnosis of ectodermal dysplasia was considered. *EDA1* sequencing, however, did not reveal any mutation. At two years of age, the patient developed several foci of painful nodular lesions in the tibia and humerus. Biopsy revealed periosteal elevation and subcortical inflammatory infiltrate

(Figure S1D). Fundoscopic exam revealed papilledema, and involvement of the retina and choroid and MRI and imaging confirmed inflammation of the optic nerve (Figure S1B, F) and leptomeninges (FigureS1G). Ongoing chorioretinitis has been treated with intraocular steroids which has progressed to include choroidal neovascular membrane formation. At two years of age, clinical immunophenotyping revealed hypogammaglobulinemia IgG 355 (453-916 mg/dL), IgA 29 (20-100 mg/dL), IgM 46 (19-146 mg/dL) with normal B cell numbers, and reduced levels of $CD4^+$ and $CD8^+$ memory T cells. Analysis of cerebrospinal fluid also revealed elevated levels of immunoglobulin. Autoantibody testing of CSF fluid was negative. Serum cytokines including IL-12 (p70), IFN-γ, and IL-6, as well as IP-10, an interferon-responsive chemokine, were elevated compared to healthy control (HC) (Figure S1I). His lowest dose of prednisone has been approximately 0.5 mg/kg daily, and attempts to taper the dose results in recurrence of fever, panniculitis and elevated inflammatory markers. At 9 years of age, during a disease flare, the patient experienced an intracranial hemorrhagic infarct.

Given the possibility that the mother of P1 may be a germline mosaic for the exon 5 deletion, carrier status of the two sisters of P1 was evaluated. These individuals show no clinical evidence of inflammation, immunodeficiency, or skewed X-inactivation.

<u>P2 (NEMO-Δex5)</u>

P2 is a 5-year-old Caucasian male of Northern European descent referred due to persistent fever, nodular skin rash, lipodystrophy, hepatosplenomegaly, and hypogammaglobulinemia, immune mediated thrombocytopenia along with persistently elevated inflammatory markers. The patient was born with normal growth parameters following an uneventful pregnancy. Symptoms began at 3 months of age with severe anemia (Hb 7.7-8.7) and persistent thrombocytopenia. Genetic testing for pathogenic mutations causing CANDLE, *PSMB4, PSMB8, PSMB9,* Aicardi-Goutières syndrome 5, *SAMHD1,* LRBA deficiency, or NOMID, *NLRP3*, were negative. Subcutaneous nodules developed over several months that on biopsy indicated early neutrophilic panniculitis with development of lymphohistiocytic infiltrate with poorly formed granulomas dissimilar to the well-formed granuloma seen in Blau disease. Between 3 months and 6 months of age, the patient developed lymphadenopathy and fevers. At 6 months of age, he

experienced an unexplained subdural hemorrhage with an infectious workup negative for congenitally acquired neonatal infection. Subsequent brain imaging revealed signs of reduced brain volume and bilateral subdural hemorrhage, but myelination of white matter was normal for his age, although evidence of optic nerve edema was apparent on physical exam. Cognitive development appears grossly normal. No intracranial calcifications were subsequently observed on brain computed tomography. While treated with immunosuppressive therapy (Cyclosporine and high dose steroids), he developed three episodes of pneumonia when hypogammaglobulinemic (IgG 209 (453-916), IgM, 28 (19-146), IgA 24 (20-100) and lymphopenic prior to immunoglobulin replacement therapy. Lymphocyte phenotyping repeatedly indicated decreased B cells. His lowest dose of prednisone has been approximately 0.75 mg/kg daily, and any attempt to taper the dose lower results in recurrence of daily fever, skin rash and elevated inflammatory markers. He has required oral steroid pulse therapy in attempt to increase platelet counts that reached a nadir of 10,000 (250,000/μL-550,000/μL) at the age of 4.

<u>P3 (NEMO-Δex5)</u>

P3 is a 3-year-old Caucasian male of European descent with onset of disease at 4 weeks of age following an uneventful delivery and neonatal period. Symptoms commenced with appearance of a nodular erythematous rash that persisted intermittently throughout the first several years of age. Histological analyses of skin biopsy samples showed superficial and deep perivascular lymphohistiocytic infiltrate with normal dermis, multinucleated giant cells and a granulomatous infiltrate. At 4 months of age, the patient developed a left subdural hemorrhage with follow up imaging revealed diffuse superficial cortical gray matter atrophy. At 4-5 months of age, elevated white blood counts were detected with WBC 27,000 and a negative infectious and autoimmune workup. At 6 months of age, the patient was started on chloroquine for systemic inflammation, joint swelling and stiffness. At 10 months of age the patient was noted to have hepatomegaly and splenomegaly detectable clinically and by imaging and periodic fevers without an apparent infectious etiology. Evidence of anterior uveitis was detected at 14 months consisting of synechiae OD>OS which resolved with ophthalmic

steroid drops. At 2 years of age lipodystrophy was noted in the temporomandibular region and feet. At 3 years of age the patient developed lymphopenia (2,380/μL (5,500/μL-15,500/μL) and neutropenia (600/μL) The patient was treated with infliximab with partial resolution of joint symptoms, cytopenias, and facial lipodystrophy, although he continued to require low dose steroid to prevent skin and systemic inflammation.

P4 (NEMO-C417R)

Patient P4 is a 35-year-old Caucasian male of European descent diagnosed with HED-ID at the age of 4 due to infection with atypical mycobacteria which has been a recurrent complication. At the age of 30, P4 experienced an opportunistic cerebral viral infection that will be described in detail elsewhere. P4 has not exhibited clinical features consistent with autoimmune or autoinflammatory disease. Due to a single nucleotide change, P4 cells express the NEMO-C417R mutation that affects the C-terminus Zinc finger and fails to fully activate NF-κB in response to TNF and a host of TLR ligands (Figure 2A)(1) (2) (3) (4) (5).

P5 (NEMO-E391X)

NEMO-E391X belongs to the NEMO-ΔCT class of mutation described previously (6, 7) and is referred to as NEMO-ΔCT throughout. Briefly, P5 is a Caucasian male of Iberian peninsula descent who was born full term and was well until 6 months of age at which point he developed fevers and was diagnosed with pneumococcal meningitis leading to hydrocephalus necessitating placement of a ventriculoperitoneal shunt. He was diagnosed with pneumonia twice and developed an abscess in the left upper thigh. Lymphocyte phenotyping studies were notable for mild leukocytosis (WBC: 10.4K/μL [3.3-9.6 K/μL], CD3# 3402 K/μL [650-2108], CD19# 451 K/μL [47-409], with a very mild decrease in the percentage of class switched memory B cells and memory B cells 0.6% CD20/CD27 [0.7-6.3%]). He was place on monthly IVIG due to low immunoglobulin levels and He has conical shaped teeth and thick hair and sweats normally. He was diagnosed with severe eczema as an infant, and now in his teens has severely dry skin. He is developmentally delayed.

P6 (NEMO-L153R)

P6 is a Caucasian male of European descent who was born full term and developed perinatal listeria and CMV sepsis and two episodes of biopsy proven CMV colitis (8-10).. Apart from Chronic molluscum, there was no overt skin involvement. The patient experienced persistent diarrhea, failure to thrive and ulceration and nongranulomatous inflammation of esophagus, stomach ileocecum and colon. He was treated with 6MP and corticosteroids. He underwent matched HSCT and had persistent intermittent colitis.

<u>P7 (NEMO-E315A)</u>

P7 is a male of Central Asian descent with features of mild ectodermal dysplasia and enhanced susceptibility to atypical mycobacteria. Autoinflammatory disease has not been reported in P7 or other individuals with mutations in the NEMO ubiquitin binding in ABIN and NEMO (UBAN) domain (7, 11).

<u>P8 (NEMO-D113N)</u>

P8 is a Caucasian male of European descent experienced early onset sinus infections and recurrent otitis media in addition to two episodes of bacterial pneumonia. Severe diarrhea and constipation and poor growth have been present since birth. The functional effect of the mutation affects CD3 induced NF-κB activation, however TLR signaling is intact (12).

In summary, similar to previously described NEMO patients, NDAS patients exhibit laboratory evidence of impaired immune system function, with hypogammaglobulinemia the only consistent finding. Despite repeated attempts to detect potential infectious causes, with the exception of 3 episodes of uncomplicated pneumonia that resolved without IV antibiotics in P2, disseminated, severe, or opportunistic infection in P1 and P3 has not been detected. This is in contrast to other NEMO PID or NEMO autoinflammatory disease phenotypes in which individuals frequently present with severe or opportunistic infection in the first few months or years of life.

1. Liu S, et al. MAVS recruits multiple ubiquitin E3 ligases to activate antiviral signaling cascades. *eLife.* 2013;2:1-24.

2. Shifera A, and Horwitz M. Mutations in the zinc finger domain of IKK gamma block the activation of NF-kappa B and the induction of IL-2 in stimulated T lymphocytes. *Mol Immunol.* 2008;45(6):1633-45.
3. Ma CA, et al. Dendritic cells from humans with hypomorphic mutations in IKBKG/NEMO have impaired mitogen-activated protein kinase activity. *Hum Mutat.* 2011;32(3):318-24.
4. Hanson EP, et al. Hypomorphic nuclear factor-kappaB essential modulator mutation database and reconstitution system identifies phenotypic and immunologic diversity. *J Allergy Clin Immunol.* 2008;122(6):1169-77 e16.
5. Temmerman ST. Impaired dendritic-cell function in ectodermal dysplasia with immune deficiency is linked to defective NEMO ubiquitination. *Blood.* 2006;108(7):2324-31.
6. Cheng L, et al. Persistent systemic inflammation and atypical enterocolitis in patients with NEMO syndrome. *Clin Immunol.* 2009;132(1):124-31.
7. Zilberman-Rudenko J, et al. Recruitment of A20 by the C-terminal domain of NEMO suppresses NF-kappaB activation and autoinflammatory disease. *Proc Natl Acad Sci U S A.* 2016;113(6):1612-7.
8. Orange JS, et al. Deficient natural killer cell cytotoxicity in patients with IKK-gamma/NEMO mutations. *J Clin Invest.* 2002;109(11):1501-9.
9. Hanson E, et al. Hypomorphic nuclear factor-kappaB essential modulator mutation database and reconstitution system identifies phenotypic and immunologic diversity. *J Allergy Clin Immunol.* 2008;122(6):1169-77.e16.
10. Miot C, et al. Hematopoietic stem cell transplantation in 29 patients hemizygous for hypomorphic IKBKG / NEMO mutations. *Blood.* 2017.
11. Filipe-Santos O, et al. X-linked susceptibility to mycobacteria is caused by mutations in NEMO impairing CD40-dependent IL-12 production. *J Exp Med.* 2006;203(7):1745-59.
12. Devora GA, et al. A Novel Missense Mutation in the Nuclear Factor-κB Essential Modulator (NEMO) Gene Resulting in Impaired Activation of the NF-κB Pathway and a Unique Clinical Phenotype Presenting as MRSA Subdural Empyema. *J Clin Immunol.* 2010;30(6):881-5.

Supplemental Figure 1. Clinical and laboratory features of NDAS (A) Conical teeth, a feature of ectodermal dysplasia in P1 (left). The teeth of P2 (middle) and P3 (right) appear normal. Hair in P1-P3 is present and appears normal. (B) chorioretinitis with choroidal neovascular membrane formation in P1 (C) hepatosplenomegaly (D,E) chronic recurrent osteomyelitis, H and E stain and radiograph depicting punched-out cortical bone defect (F,G) white matter changes with leptomeningeal enhancement. (H) CNS cortical atrophy in P2. (I) Serum cytokine levels from patient P1 and healthy control drawn on two different dates separated 12 months apart. #: level below limits of detection (J) Left, Sashimi plots depict quantitative splice junctions from alignment data of whole blood RNA-Seq from NDAS patient and HC. Right, ratio of alternatively spliced to exon4-5 and exon5-6 splice junctions from 3 NDAS patients and 30 healthy controls representative of Sashimi plots shown. (K) Full length and NEMO-∆ex5 mRNA isoform quantitation by qPCR from P1, P2, P3 and HC skin fibroblasts. (L) Frequency of

reference bases for *IKBKG* mutation sites observed in a large healthy subject cohort compared to patient genotypes. Unfiltered reads from a male and female healthy cohort and patients are plotted as normalized bases for individuals by column with bases colored by identity. A. Rate of observed base calls overlapping the two homologous sites corresponding to the G>A missense mutation in P1 in the *IKBKG* gene. Patient whole genome sequence was unavailable for this locus. B and C correspond to the T>G mutation in P2 and the G>A mutation in P3 near the *IKBKG* gene, respectively. (M) whole blood RNA-Seq NF-κB-response gene expression profile from P1, P2 and P3 taken during serial visits for P1 and P3, compared to HC (n=13) and NOMID autoinflammatory disease control (n=10) patients.

Supplemental Fig 2. NEMO-Δex5 which fails to associate with TBK1 renders cells susceptible to hPIV3-GFP infection, although TNF-induced NF-κB activation and poly(I:C)-induced IRF-3 phosphorylation are intact (A) HEK 293T cells were transfected with NEMO mutant forms and NEMO was immunoprecipitated; association with TBK1 was assayed by Western blot. (B) P1 and HC skin fibroblasts were stimulated with poly(I:C) in a timecourse as indicated and Western blot of whole cell lysates was done to detect p-IRF3, NEMO and ACTIN controls. (C) P1 and healthy control skin fibroblasts were stimulated with LPS 10 ng/mL for 60 minutes and stained for NF-κB subunit p65 by immunofluorescence microscopy (20X magnification), quantitation of p65 nuclear intensity is shown to the right, unpaired t test. The same image of unstimulated p65-stained cells is shown in Figure 2E. (D) P1 and HC skin fibroblasts stimulated with LPS 10ng/mL for 3 hours with gene expression measured by RNA-Seq. Shown is average fold induction of NF-κB response genes, n=3. Red dashed lines indicate 3-fold limit. (E) Gene Set Enrichment Analysis (GSEA) of P1/HC from RNASeq on LPS stimulated skin fibroblasts as in D. (F) NEMO deficient Jurkat T cells were reconstituted with full length NEMO, or NEMO-Δex5 and stimulated with TNF or rhodamine tagged poly(I:C) for 9 hrs. or 18 hours respectively, NF-κB reporter activity was measured by surface staining rat. Thy1, quantitation of NF-κB reporter positive cells, of n=4 is right. (G) NEMO deficient 293T cells were reconstituted with full length NEMO or NEMO-Δex5 and stimulated with TNF 20ng/mL or poly(:C) 50μg/mL in a time course as indicated, Western blot of NF-κB activation (phosphor-p65 S536 was determined by flow cytometry, NEMO and loading control. (H) iPS-derived fibroblast-like cells from NDAS (P1), and other NEMO (with autoinflammatory disease and NEMO UBAN domain mutation) were infected with hPIV3-GFP and viral protein expression was measured by acquiring serial images using brightfield and green filters for GFP. *, P < 0.05; **, P < 0.01; ***, P < 0.001, RM 2-way ANOVA.

Supplemental Figure 3. Monocytes and T cells expressing NEMO-Δex5 are responsive to poly(I:C) stimulation and produce an excess of IFN. (A) IPA gene pathway analysis of whole blood RNA-Seq from three separate P1 samples drawn at serial clinic visits compared to age matched healthy control showing differentially regulated pathways. (B) GSEA of whole blood RNASeq indicates IFNα and IFNγ pathway enrichment in PBMC from NDAS patients P1 and P3 compared to non-NDAS NEMO patients that exhibit a downregulated TNFR signaling expression profile. (C) "3-gene NF-kB/STAT1score" and an "11-gene NF-kB-only validation score" plotted versus NEMO-Dex5 alternative splicing ratio as done in 3C. (D) PBMC from NDAS patients,

NEMO control and healthy control were isolated, rested for 2 hours and incubated with Brefeldin A for 4 hours, fixed, and stained for surface marker expression in addition to intracellular IFNγ, right, quantitation. (E) Freshly isolated PBMC were briefly rested and left in media or stimulated with anti-CD3/CD28 coated beads or poly(I:C). Cell supernatants were collected after 12 hours and concentrations of secreted cytokines were analyzed by bead capture assay. (F) stable THP1 NEMO mutant lines were stimulated with HMW transfected poly(I:C) for 30 minutes or 60 minutes followed by fixation and staining with specific antibodies to IκBα and phospho-p65 S536 respectively. (G) NEMO-Δex5 or full-length NEMO retrovirally expressed in NF-κB reporter Jurkat T cells were stimulated with LMW poly(I:C) for 18 hours, fixed and stained to detect surface expression of NF-κB reporter. *, $P < 0.05$; **, $P < 0.01$; ***, $P < 0.001$, unpaired t test unless otherwise stated.

Supplemental Figure 4.
293T control IKKi overexpression
(A) Lentiviral driven IKKi expression in iPSC-fibroblast-like MSC was detected following Western blot of whole cell lysates using antibody specific for IKKi with IκBα as a loading control. (B) Quantitation of IRES-mCherry in cells from the experiment shown in Figure 5F transduced with empty vector or IKKi.

Table S1 – GSEA NDAS vs HC, NDAS vs. 'other NEMO', and 'other NEMO' vs NDAS

NDAS vs. HC

	GS	SIZE	NES	NOM p-val	FDR q-val
1	HALLMARK_MYC_TARGETS_V1	197	0.48	1.73	0.000
2	HALLMARK_WNT_BETA_CATENIN_SIGNALING	42	0.55	1.60	0.002
3	HALLMARK_PROTEIN_SECRETION	95	0.47	1.58	0.000
4	HALLMARK_MYC_TARGETS_V2	58	0.47	1.45	0.029
5	HALLMARK_PEROXISOME	103	0.42	1.43	0.010
6	HALLMARK_INTERFERON_GAMMA_RESPONSE	197	0.40	1.43	0.000
7	HALLMARK_COMPLEMENT	195	0.39	1.39	0.006
8	HALLMARK_INTERFERON_ALPHA_RESPONSE	93	0.41	1.35	0.023
9	HALLMARK_INFLAMMATORY_RESPONSE	197	0.36	1.33	0.011

NDAS vs 'other NEMO'

	GS	SIZE	NES	NOM p-val	FDR q-val
1	HALLMARK_INTERFERON_ALPHA_RESPONSE	93	2.00	0.000	0.001
2	HALLMARK_MYC_TARGETS_V1	197	1.59	0.000	0.020
3	HALLMARK_OXIDATIVE_PHOSPHORYLATION	183	1.58	0.000	0.014
4	HALLMARK_INTERFERON_GAMMA_RESPONSE	197	1.56	0.000	0.014
5	HALLMARK_PROTEIN_SECRETION	95	1.35	0.034	0.097
6	HALLMARK_WNT_BETA_CATENIN_SIGNALING	42	1.33	0.062	0.105
7	HALLMARK_MYC_TARGETS_V2	58	1.31	0.070	0.104
8	HALLMARK_PEROXISOME	103	1.28	0.048	0.130

'other NEMO' vs NDAS

	GS	SIZE	NES	NOM p-val	FDR q-val
1	HALLMARK_TNFA_SIGNALING_VIA_NFKB	198	-1.95	0.000	0.000
2	HALLMARK_EPITHELIAL_MESENCHYMAL_TRANSITION	196	-1.55	0.000	0.030
3	HALLMARK_APICAL_SURFACE	43	-1.37	0.055	0.171

4	HALLMARK_APOPTOSIS	159	-1.32	0.027	0.225

Figure S1

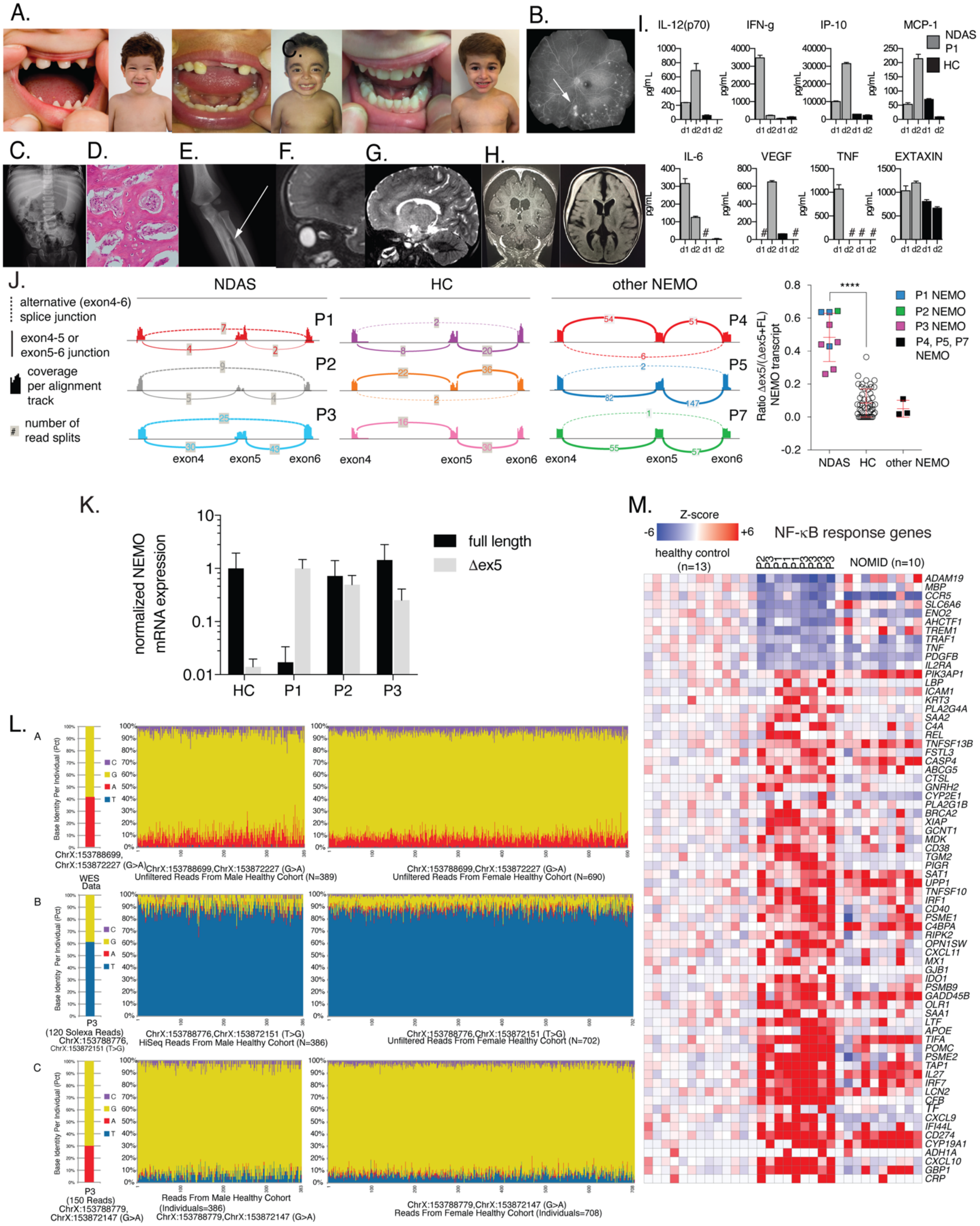

Supplemental Figure 1. Clinical and laboratory features of NDAS (A) Conical teeth, a feature of ectodermal dysplasia in P1 (left). The teeth of P2 (middle) and P3 (right) appear normal. Hair in P1-P3 is present and appears normal. (B) chorioretinitis with

choroidal neovascular membrane formation in P1 (C) hepatosplenomegaly (D,E) chronic recurrent osteomyelitis, H and E stain and radiograph depicting punched-out cortical bone defect (F,G) white matter changes with leptomeningeal enhancement. (H) CNS cortical atrophy in P2. (I) Serum cytokine levels from patient P1 and healthy control drawn on two different dates separated 12 months apart. #: level below limits of detection (J) Left, Sashimi plots depict quantitative splice junctions from alignment data of whole blood RNA-Seq from NDAS patient and HC. Right, ratio of alternatively spliced to exon4-5 and exon5-6 splice junctions from 3 NDAS patients and 30 healthy controls representative of Sashimi plots shown. (K) Full length and NEMO-Δex5 mRNA isoform quantitation by qPCR from P1, P2, P3 and HC skin fibroblasts. (L) Frequency of reference bases for *IKBKG* mutation sites observed in a large healthy subject cohort compared to patient genotypes. Unfiltered reads from a male and female healthy cohort and patients are plotted as normalized bases for individuals by column with bases colored by identity. A. Rate of observed base calls overlapping the two homologous sites corresponding to the G>A missense mutation in P1 in the *IKBKG* gene. Patient whole genome sequence was unavailable for this locus. B and C correspond to the T>G mutation in P2 and the G>A mutation in P3 near the *IKBKG* gene, respectively. (M) whole blood RNA-Seq NF-κB-response gene expression profile from P1, P2 and P3 taken during serial visits for P1 and P3, compared to HC (n=13) and NOMID autoinflammatory disease control (n=10) patients.

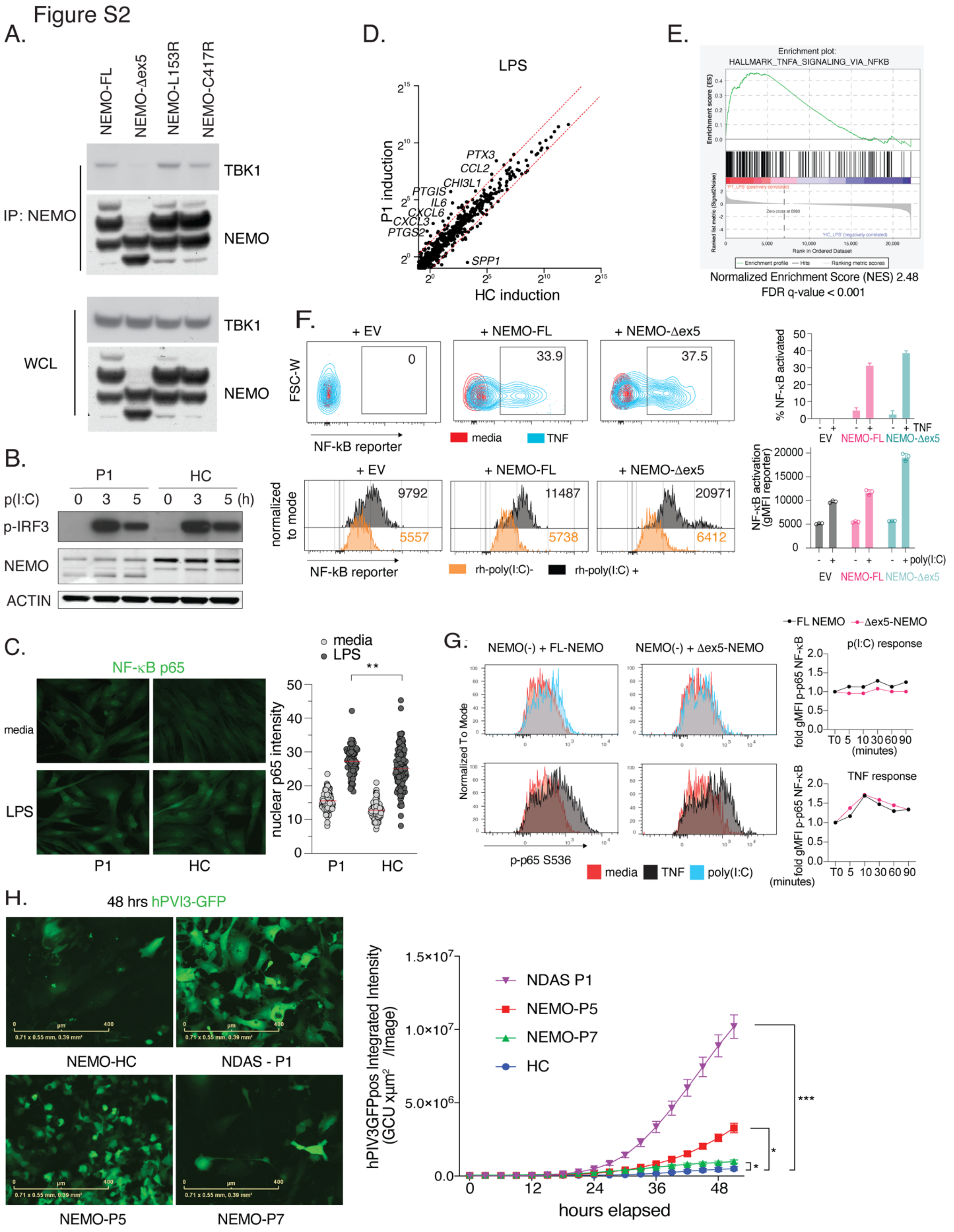

Supplemental Fig 2. NEMO-Δex5 which fails to associate with TBK1 renders cells susceptible to hPIV3-GFP infection, although TNF-induced NF-κB activation and

poly(I:C)-induced IRF-3 phosphorylation are intact (A) HEK 293T cells were transfected with NEMO mutant forms and NEMO was immunoprecipitated; association with TBK1 was assayed by Western blot. (B) P1 and HC skin fibroblasts were stimulated with poly(I:C) in a timecourse as indicated and Western blot of whole cell lysates was done to detect p-IRF3, NEMO and ACTIN controls. (C) P1 and healthy control skin fibroblasts were stimulated with LPS 10 ng/mL for 60 minutes and stained for NF-κB subunit p65 by immunofluorescence microscopy (20X magnification), quantitation of p65 nuclear intensity is shown to the right, unpaired t test. (D) P1 and HC skin fibroblasts stimulated with LPS 10ng/mL for 3 hours with gene expression measured by RNA-Seq. Shown is average fold induction of NF-κB response genes, n=3. Red dashed lines indicate 3-fold limit. (E) Gene Set Enrichment Analysis (GSEA) of P1/HC from RNASeq on LPS stimulated skin fibroblasts as in D. (F) NEMO deficient Jurkat T cells were reconstituted with full length NEMO, or NEMO-Δex5 and stimulated with TNF or rhodamine tagged poly(I:C) for 9 hrs. or 18 hours respectively, NF-κB reporter activity was measured by surface staining rat. Thy1, quantitation of NF-κB reporter positive cells, of n=4 is right. (G) NEMO deficient 293T cells were reconstituted with full length NEMO or NEMO-Δex5 and stimulated with TNF 20ng/mL or poly(:C) 50μg/mL in a time course as indicated, Western blot of NF-κB activation (phosphor-p65 S536 was determined by flow cytometry, NEMO and loading control. (H) iPS-derived fibroblast-like cells from NDAS (P1), and other NEMO (with autoinflammatory disease and NEMO UBAN domain mutation) were infected with hPIV3-GFP and viral protein expression was measured by acquiring serial images using brightfield and green filters for GFP. *, $P < 0.05$; **, $P < 0.01$; ***, $P < 0.001$, RM 2-way ANOVA.

Figure S3

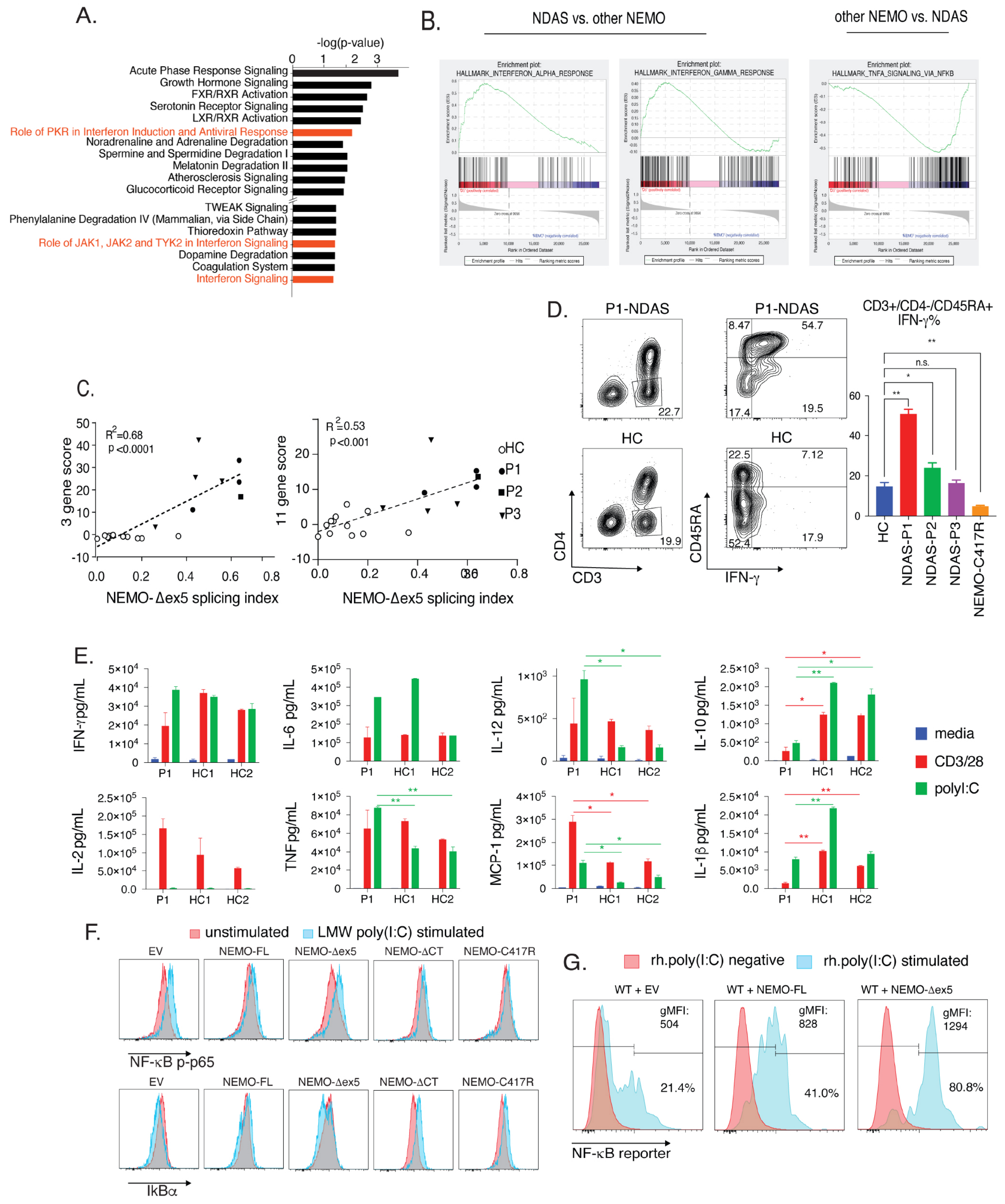

Supplemental Figure 3. Monocytes and T cells expressing NEMO-Δex5 are responsive to poly(I:C) stimulation and produce an excess of IFN. (A) IPA gene

pathway analysis of whole blood RNA-Seq from three separate P1 samples drawn at serial clinic visits compared to age matched healthy control showing differentially regulated pathways. (B) GSEA of whole blood RNASeq indicates IFNα and IFNγ pathway enrichment in PBMC from NDAS patients P1 and P3 compared to non-NDAS NEMO patients that exhibit a downregulated TNFR signaling expression profile. (C) "3-gene NF-kB/STAT1score" and an "11-gene NF-kB-only validation score" plotted versus NEMO-Dex5 alternative splicing ratio as done in 3C. (D) PBMC from NDAS patients, NEMO control and healthy control were isolated, rested for 2 hours and incubated with Brefeldin A for 4 hours, fixed, and stained for surface marker expression in addition to intracellular IFNγ, right, quantitation. (E) Freshly isolated PBMC were briefly rested and left in media or stimulated with anti-CD3/CD28 coated beads or poly(I:C). Cell supernatants were collected after 12 hours and concentrations of secreted cytokines were analyzed by bead capture assay. (F) stable THP1 NEMO mutant lines were stimulated with HMW transfected poly(I:C) for 30 minutes or 60 minutes followed by fixation and staining with specific antibodies to IκBα and phospho-p65 S536 respectively. (G) NEMO-Δex5 or full-length NEMO retrovirally expressed in NF-κB reporter Jurkat T cells were stimulated with LMW poly(I:C) for 18 hours, fixed and stained to detect surface expression of NF-κB reporter. *, $P < 0.05$; **, $P < 0.01$; ***, $P < 0.001$, unpaired t test unless otherwise stated.

Figure S4

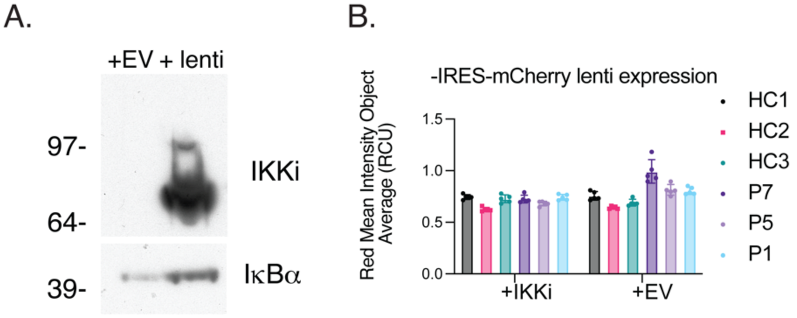

Supplemental Figure 4.

293T control IKKi overexpression

(A) Lentiviral driven IKKi expression in iPSC-fibroblast-like MSC was detected following Western blot of whole cell lysates using antibody specific for IKKi with IκBα as a loading control. (B) Quantitation of IRES-mCherry in cells from the experiment shown in Figure 5F transduced with empty vector or IKKi.
